# Next-generation newborn screening: feasibility of combined genetic and biochemical testing for 95 treatable inherited metabolic disorders

**DOI:** 10.1007/s11306-026-02486-6

**Published:** 2026-06-25

**Authors:** Abigail Veldman, C. Edel Arar, M. B. Gea Kiewiet, Els Voorhoeve, Martijn E. T. Dollé, Monique de Sain-van der Velden, Rose E. Maase, Francjan J. van Spronsen, Birgit Sikkema-Raddatz, M. Rebecca Heiner-Fokkema

**Affiliations:** 1 Division of Metabolic Diseases, Beatrix Children’s Hospital, University Medical Center Groningen, University of Groningen, Groningen, The Netherlands; 2 Department of Genetics, University Medical Center Groningen, University of Groningen, Groningen, The Netherlands; 3 Centre for Health Protection, National Institute for Public Health and the Environment, Bilthoven, The Netherlands; 4 Section Metabolic Diagnostics, Department of Genetics, University Medical Centre Utrecht, Utrecht, The Netherlands; 5 Laboratory of Metabolic Diseases, Department of Laboratory Medicine, University Medical Center Groningen, University of Groningen, Groningen, The Netherlands

**Keywords:** Newborn screening, Dried blood spot, Next-generation sequencing, Genomic screening, DNA-based screening, Biochemical testing, Biomarkers, Inherited metabolic diseases

## Abstract

**Introduction:**

Next-generation sequencing (NGS) is gaining attention in newborn screening (NBS) for its ability to detect treatable genetic disorders, especially those without a biochemical footprint. However, NGS-NBS requires interpreting variants without phenotype information or family trio analysis. Biochemical tests, preferably in dried blood spots (DBS), are therefore useful to confirm the pathogenicity of variants identified by NGS-NBS and increase its specificity and sensitivity.

**Objectives:**

We aimed to explore the potential of combined genetic-biochemical testing for 95 treatable Inherited Metabolic Disorders (IMD) considered eligible for NGS-NBS (100 genes) previously identified by our research group.

**Methods:**

We reviewed the Collaborative Laboratory Integrated Reports (CLIR) and carried out systematic literature reviews in PubMed and Embase to identify biochemical tests for 95 IMD. Biochemical tests conducted on DBS were differentiated from tests that require referral.

**Results:**

We identified DBS-biochemical tests for 72 of the 95 IMD (77/100 genes). DBS-based biochemical tests for 55 IMD (60 genes) are already implemented in NBS. For the other 23 IMD, biochemical tests in non-DBS specimens are reported, although some are less sensitive when measured at neonatal age in presymptomatic infants.

**Conclusion:**

We present a comprehensive overview of current biochemical tests for 95 IMD. These tests can be used to confirm inconclusive NGS-NBS results, and combined genetic-biochemical testing is expected to improve both the negative and positive predictive values of NBS programs.

**Supplementary Information:**

The online version contains supplementary material available at https://doi.org/10.1007/s11306-026-02486-6.

## Introduction

Newborn screening (NBS) programs aim to identify (inherited) disorders in neonates as early as possible to enable early therapeutic interventions that may prevent or reduce severe disability or morbidity or avert mortality (Therrell et al., 2024). Current NBS programs are predominantly based on biochemical tests that measure enzyme activities or biomarker concentrations in dried blood spots (DBS). The current Dutch NBS program, which includes 27 disorders, has been highly successful, showing a sensitivity of 98.0% and a participation rate of 98.7% in 2024 (TNO, 2025). The biochemical NBS for inherited metabolic disorders (IMD), in particular, has had a sensitivity of 100% over the past few years, but its positive predictive values (PPV) range between 7.0% and 100%, with a mean PPV of 40.6% (TNO, 2025). Expansion of biochemical testing in NBS can, however, be hampered by the lack of sensitive and specific DBS biomarkers for many treatable (inherited) disorders. In addition, the DBS source material is finite and may become a limiting factor as new diseases are added to NBS programs. This is especially true if multiple-tier testing algorithms are required, as is the case for the Dutch screening on cystic fibrosis (Bouva et al., 2024).

Genetic analyses based on next-generation sequencing (NGS) methods are expected to revolutionize NBS and, therefore, are being increasingly investigated worldwide (Minten et al., 2025). NGS holds the potential to include most genetic disorders for which there are no means to evaluate the biochemical footprint in NBS DBS. It requires only a single DBS for analysis (Kiewiet et al., 2024). Another advantage of genetic methods is that, unlike many metabolites or enzyme activities, DNA is both more stable over time and mostly not susceptible to external factors like (maternal) nutrition, (gestational) age, or (maternal) vitamin deficiencies, that cause some of the false-positive and false-negative results in the current biochemical NBS (Meijer et al., 2025). In addition, abnormal metabolites detected in NBS may reflect a metabolic disorder in the mother rather than in the newborn (van Spronsen et al., 2021).

Although the implementation of NGS in NBS holds great potential, challenges remain. Despite the promising results from several large-scale genetic NBS (NGS-NBS) studies (Boemer et al., 2025; Chen et al., 2023; Green et al., 2023; Kaplanis et al., 2025; Ziegler et al., 2024), which have included thousands of newborns, the translation from research into nationwide public health programs remains difficult due to several factors, including, but not limited to, challenges in variant interpretation, particularly in pre-symptomatic individuals.

Reporting variants of unknown significance (VUS) in NGS-NBS remains controversial (Kemp et al., 2023). Most recent studies adopting an NGS-only approach have decided not to report VUS to minimize false-positive results. However, excluding VUS reduces sensitivity and, consequently, lowers the negative predictive value (NPV) compared to the current biochemical test-based NBS programs (Bick et al., 2025), which is a known limitation of current short-read NGS techniques (Bodian et al., 2016). These data together indicate that although NGS-NBS enables the identification of a broader range of genetic disorders, more affected individuals will be missed compared to the current biochemical NBS. In addition, more neonates may be unnecessarily referred when using an NGS-only strategy, making the justification of a complete replacement of the current biochemical NBS with NGS-NBS difficult.

Despite its considerable potential, the implementation of NGS in NBS is further shaped by ethical, technical, and financial challenges, including decisions on which disorders should be included in screening panels (Minten et al., 2025). The Wilson and Jungner (W&J) principles (J.M.G. Wilson, 1968) provide some guidance, but are limited in the genomic era, as they were not designed for probabilistic genetic findings with variable expressivity, incomplete penetrance, and heterogeneous age of onset and phenotypic severity (Downie et al., 2026). This raises ethical concerns, especially around overdiagnosis and the “right not to know”, particularly for results without immediate childhood clinical utility. This is particularly relevant in a genomics-only NBS approach. In anticipation of these challenges, our research group previously proposed a list of 95 treatable IMD that we considered eligible for NGS-NBS (Veldman et al., 2024) focusing on early treatability. Although NGS offers some significant advantages for NBS, the challenges associated with NGS-NBS need to be balanced against the most important fundamental principle for NBS, continuing to do good and avoiding harm. In NBS, ‘harm’ can include unnecessary follow-up and overtreatment at the individual level, as well as preventing potential destabilization of an already successful and trustworthy public health program on a societal scale. When considering the incorporation of NGS in NBS, avoidance of additional harms is as important as the potential benefits offered by NGS (Bick et al., 2022).

At present, a combined approach integrating the strengths of both genetic and biochemical testing is potentially feasible for the IMD already included in the Dutch NBS program (Fecarotta et al., 2025). The feasibility of a combined approach for other genetic disorders will depend on the availability of a biomarker that can reflect the underlying genetic condition through the presence or absence of metabolites or enzyme activities in blood or other bodily fluids. Ideally, such a biomarker should be detectable in DBS, to allow both tests to be performed on the same DBS without the need for referral for additional sampling. Because pathogenic variants in most IMD-associated genes typically result in abnormal metabolite profiles, biochemical testing can also be used to support or refute the pathogenicity of detected variants. To this purpose, i.e., to explore the potential of combined genetic-biochemical testing to improve both the sensitivity and specificity of NBS programs, we conducted a review of available biochemical tests in DBS and other biological material for the 95 treatable IMD we identified previously (Veldman et al., 2024).

## Materials and methods

### Definition of biomarkers

In our study, we defined a biological marker or biomarker as a measurable biological indicator (including enzyme activity assays and other biochemical tests) used to detect the presence and severity of a disease or a secondary condition. Our primary aim was to identify biomarkers in DBS because this allows a combined biochemical and genetic testing screening approach within the current NBS setting. However, our search was also extended to biomarkers found in blood or blood components (e.g., serum, plasma, erythrocytes, leukocytes), cerebrospinal fluid (CSF), urine, dried urine spots, feces, biopsies of all types of tissue, connective tissue cells (e.g., fibroblasts), immune cells, and saliva. The disorder-specificity of a biomarker was a preferred but not an absolute prerequisite because, in practice, abnormal biochemical features on their own or in combination with a (likely) pathogenic ((L)P) variant or a VUS, will be sufficient reason for referral. Therefore, to ensure the highest NPVs (i.e., to prevent “missed” patients), we deemed biomarker sensitivity most important.

Based on the amount and consistency of available data, we classified the DBS biomarkers found in the database review and the systematic literature review into four categories:

- Class I ─ biomarkers or biochemical tests already used by one of the NBS programs worldwide.
- Class II ─ biomarkers or biochemical tests as either a secondary finding in a NBS program or with only a few cases reported.
- Class III ─ biomarkers or biochemical tests measurable in DBS but never reported in patients with the specific IMD.
- Class 0 ─ no biomarkers or biochemical tests in DBS were reported.

Data on sensitivity and the total number of IMD cases (true positives) were included in the results when available (Online Appendix A).

### Study design

**Fig. 1:**
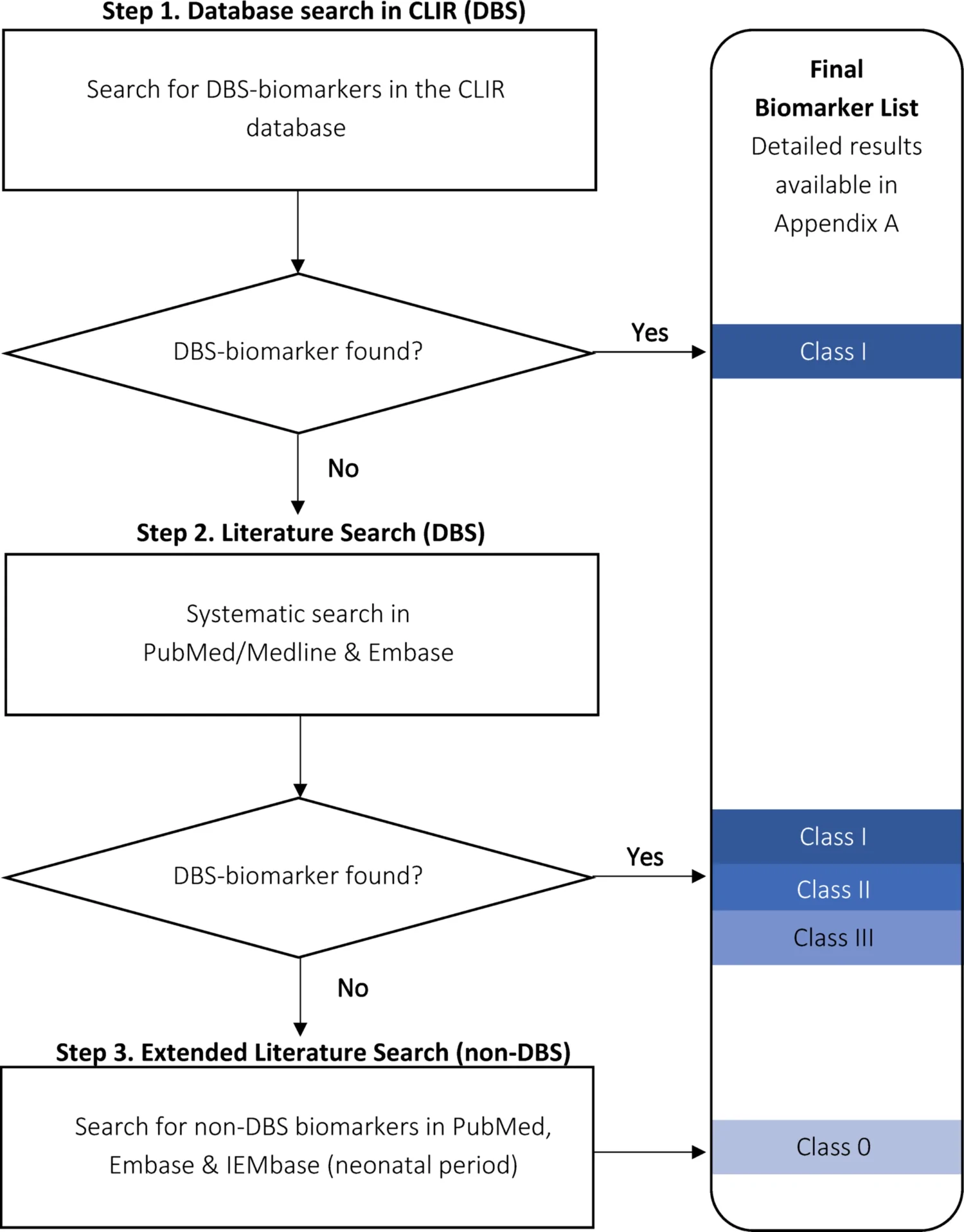
Combined search strategy to identify biomarkers in dried blood spots (DBS) and other biological materials in the 95 Inherited Metabolic Disorders (IMD) (100 genes) from Veldman et al. (2024). CLIR: Collaborative Laboratory Integrated Reports. IEMbase.com: Inborn Errors of Metabolism Knowledgebase

The list of 100 genes for 95 treatable IMD was previously established in a Dutch preparatory study exploring next-generation sequencing as a first-tier approach in NBS (NGSf4NBS) (Veldman et al., 2024). These 100 genes were selected based on the W&J principles (J.M.G. Wilson, 1968). 42 Of these genes are associated with the 20 IMD included in the 2026 Dutch NBS program, representing either the primary targeted IMD or non-targeted IMD resulting from abnormal biochemical tests (Veldman et al., 2024). Our review was conducted in three steps (see Fig. 1), and a more detailed description of the steps is provided in Method Sect. 2.3., 2.4. and 2.5. Complete search strategies and prompts to replicate the data are provided in Online Appendix B.

### Step 1. Review of the collaborative laboratory integrated reports database

We first consulted the Collaborative Laboratory Integrated Reports (CLIR) database (Biochemical Genetics Laboratory et al. 2026). This database is an interactive web tool created by the Biochemical Genetics Laboratory, the Department of Laboratory Medicine and Pathology, and the Department of Information Technology of the Mayo Clinic. CLIR includes a ‘Post Analytical Tools’ functionality, which is a database in which NBS data from biochemical tests conducted on NBS DBS are combined with four neonate covariates (biological sex, gestational age, birth weight and age at heel prick). These data are gathered from NBS programs all over the world (Biochemical Genetics Laboratory et al. 2026). Using the CLIR database, we identified IMD with available biochemical tests from existing NBS programs. Specific settings within CLIR allow restriction of the output to biomarkers that are considered informative for each IMD, i.e., biomarkers for which no overlap is observed between concentrations in true-positive NBS referrals and those in the reference (healthy) population within the available CLIR datasets. This results in biomarkers showing apparent complete separation in these cohorts. These CLIR biomarkers have demonstrated very high sensitivity in reported datasets; however, whilst these findings are based on clinically confirmed cases detected via NBS, the precise definition of each clinically confirmed case is unknown. For this reason, the generalizability of the conclusions drawn from the data in CLIR for population-based screening becomes more robust with the number of individual cases for each IMD. The prompts to replicate these data are provided in Online Appendix B. The chart with the box plots for each IMD was exported into a PDF file and saved in a shared database. Online Appendix A lists all the identified biomarkers from CLIR, and all are classified as Class I.

### Step 2. Systematic literature review to identify DBS biomarkers in PubMed and Embase

We performed a systematic literature review in PubMed (National Library of Medicine, United States) and Embase (Elsevier, Europe) to identify DBS biomarkers only for the IMD not present in the CLIR database (Biochemical Genetics Laboratory et al. 2026), assuming that CLIR contains data on all available informative DBS markers. Here, we aimed to identify at least one publication in which a biomarker is described (and preferably tested in DBS). A comprehensive PubMed search strategy was combined with a query tailored to each of the remaining IMD, as detailed in Online Appendix B. No filters or limits were applied. When a search yielded more than 50 records, a third more specific query was added (see Online Appendix B). The titles and abstracts screening included studies published in English with full text accessibility. If the report’s title or abstract did not concern the intended IMD or the relevant topic, the report was excluded from further review. In addition, screening for duplicates was performed. Full-text reports were then assessed for eligibility based on two primary inclusion criteria: (1) IMD in scope of study, but not included in CLIR database and (2) description of a biomarker or biochemical test using DBS.

If, for an IMD, no DBS biomarker for an IMD was retrieved from PubMed, Embase (Elsevier, EU) was consulted to increase the overall coverage of our search. We used the query translator tool in Embase to translate our PubMed query into an Embase search strategy. The same inclusion and exclusion criteria used in PubMed were applied while evaluating the abstracts and full-text reports. All the DBS biomarkers identified this way are included in Online Appendix A and received a classification. If a non-DBS biomarker was found during the search for a DBS biomarker, these results were included in a separate column in Online Appendix A to assist Step 3 (see below). To ensure the quality and transparency of our systematic literature review for DBS biomarkers, we applied the guidelines of the PRISMA 2020 statement (Page et al., 2021).

### Step 3. An expanded search strategy to identify biomarkers in alternative biological matrices beyond DBS (non-DBS)

If no DBS biomarker for an IMD was identified in Steps 1 and 2, we applied a more detailed search strategy using PubMed and Embase to identify a biomarker in non-DBS matrices. The records describing non-DBS biomarkers identified during Step 2 were complemented by an additional targeted search. The search terms used were a combination of the IMD including synonyms, and the name of the gene. If this search yielded more > 50 results, the search was narrowed using Medical Subject Headings (MeSH) terms related to biological matrices, including blood and blood components, immune cells, saliva, urine, feces, (tissue) biopsy, CSF, and fibroblasts.

Lastly, we consulted the Inborn Errors of Metabolism Knowledgebase (IEMbase) (Ferreira et al., 2019) to check for non-DBS biomarkers that may not have been captured in Steps 2 and 3. IEMbase is an online knowledgebase system using Artificial Intelligence (AI) designed for curation and diagnostic support of IMD (Ferreira et al., 2019). As this resource provides biomarker information for non-DBS materials without associated primary references, it was used only as a complementary source. Only biomarkers reported as elevated or decreased during the neonatal period from birth to one month of age were included.

### Strategy to minimize the risk of bias

Two independent researchers (A.V., M.R.H-F.) conducted the literature review to minimize selection and reviewer bias. Afterwards, all records in which a biomarker was identified were discussed and reviewed jointly. Search strategies were evaluated to ensure that all recent and relevant literature had been adequately captured. Online Appendix A was permitted to be supplemented with additional sources, provided they met the predefined inclusion criteria. Risk of bias was minimized by setting clear inclusion criteria and by following the PRISMA 2020 guidelines (Page et al., 2021). Since we aimed to find relevant publications with evidence of a DBS biomarker, rather than to perform an exhaustive literature review, no formal strategies were implemented to address publication bias, language bias, and citation bias.

## Results

### Results from the CLIR database

**Fig. 2:**
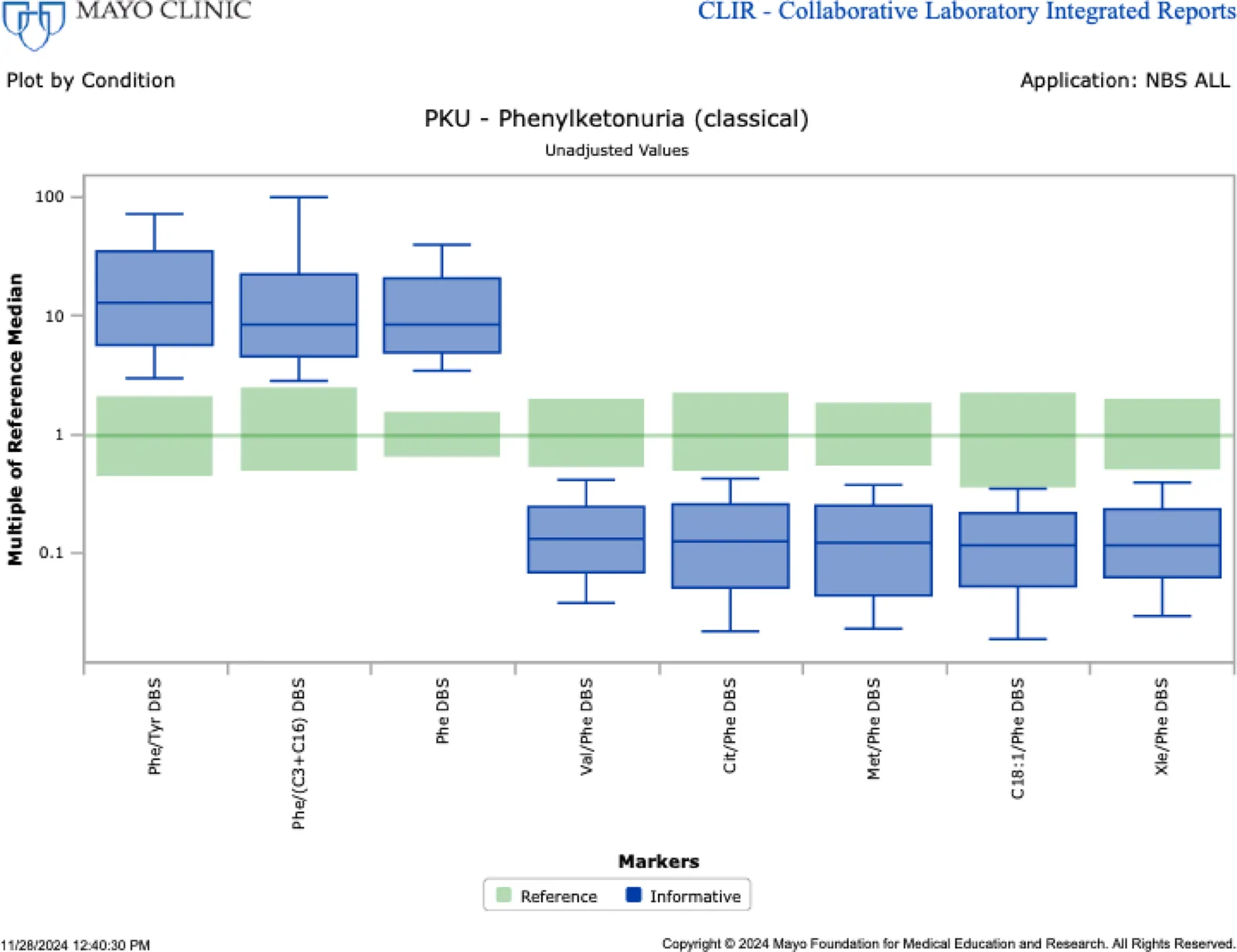
An example boxplot of DBS biomarkers for one of the 45 inherited metabolic disorders (50 genes) retrieved from CLIR. Phenylketonuria (PKU), based on *n* = 1565 patients. Phe = Phenylalanine, Tyr = Tyrosine, C3 = Propionylcarnitine, C16 = C16-carnitine, Val = Valine, Cit = Citrulline, Met = Methionine, C18:1 = Oleoylcarnitine, Xle = Total leucine (Ile, Leu, Allo-Ile)

Data collection from the CLIR database was conducted between 1 December 2024 and 1 January 2025. The review of the CLIR database identified biomarkers in DBS for 45 IMD that are linked to 50 genes. Using the settings described in Online Appendix B, box plots were generated corresponding to each IMD. Figure 2. shows the boxplot for phenylketonuria (PKU) (*PAH*, MIM *612349, #261600) as example. An overview of the 45 IMD (50 genes) with available DBS-biomarkers retrieved from CLIR, including the number of reported cases, is provided in Online Appendix A.

### Systematic literature review of PubMed/Medline and Embase to identify DBS biomarkers

#### DBS biomarkers

**Fig. 3:**
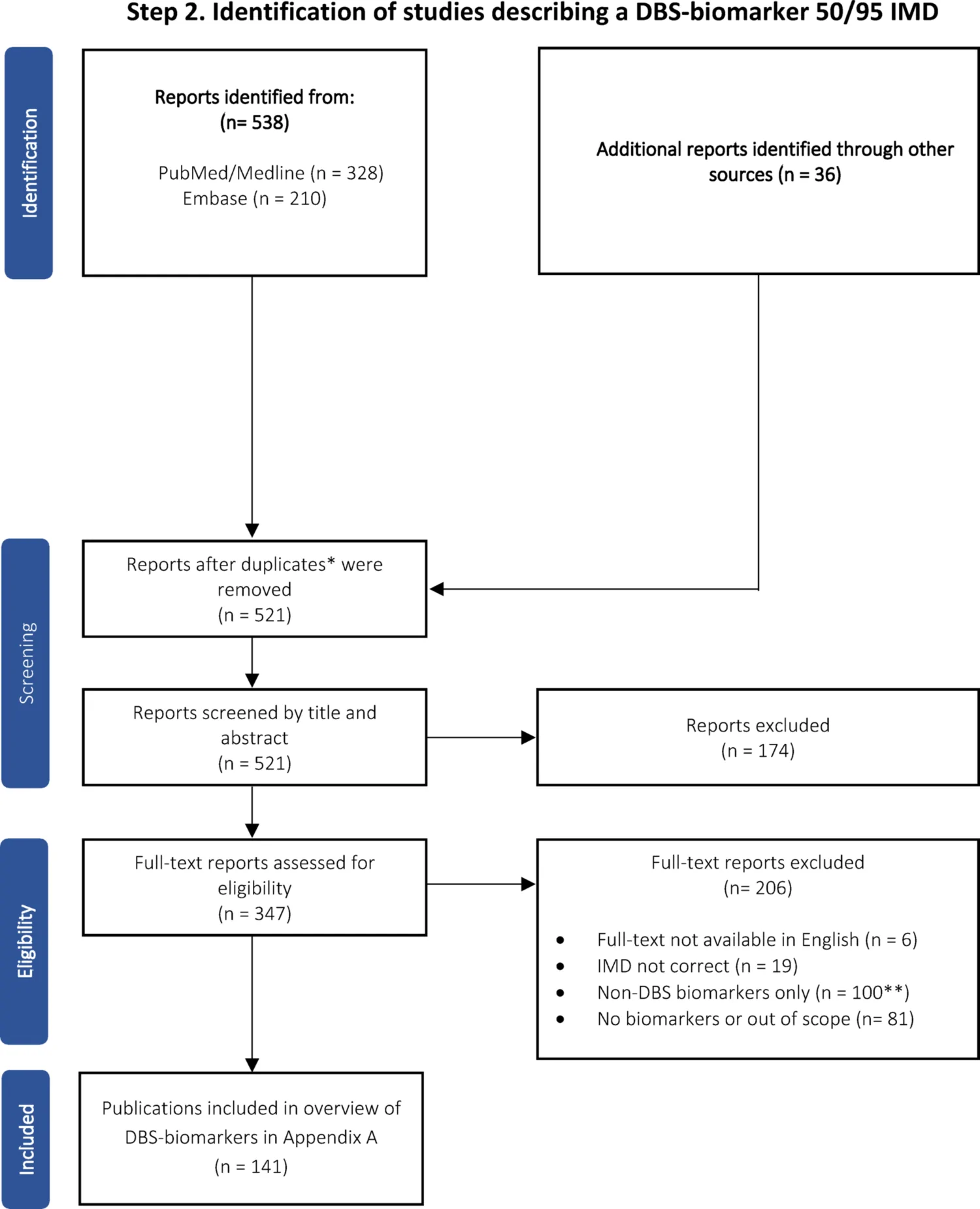
PRISMA flow diagram of the systematic literature review for DBS-biomarkers. DBS: Dried blood spot, IMD: Inherited metabolic disorder * As every of the 50 IMD were reviewed individually, only duplicate reports for the same IMD (*n* = 53) were removed. ** During the DBS-search there were 100 reports containing non-DBS biomarkers, these were complemented with the results from Step 3

Following the identification of DBS biomarkers for 45 IMD (50 genes) in the CLIR database, we conducted a literature review to search for DBS biomarkers for the remaining 50 IMD (50 genes). The initial PubMed search yielded 328 reports, while an Embase search added 210 reports; 36 additional reports were derived from cross-references or from our reference repositories, totaling 574 reports. For each gene, the number of results varied from 3 to 40 reports. After removing 53 duplicates and excluding 174 reports based on title and abstract, 347 full-text articles were assessed. Of these, 206 were excluded according to the predefined criteria. Of these 206 excluded reports, 100 reports contained non-DBS biomarker data (documented in Step 3). A total of 141 articles, reporting on a DBS biomarker, were added to the overview in Online Appendix A. For 27 IMD (27 genes) of the 50 IMD, at least one biomarker in DBS was identified. Of these 27 IMD, 10 IMD were classified as Class I, 11 as Class II, and 6 as Class III. The systematic review was conducted from 1 January 2025 until 1 April 2025. See Fig. 3. for the PRISMA flowchart of this systematic literature review.

#### Non-DBS biomarkers

For the remaining 23 IMD (23 genes) without DBS biomarkers, at least one biomarker was identified in non-DBS material, either from the initial search or an additional targeted search in PubMed and Embase. For these 23 IMD, the child should be referred after a positive NGS-based NBS result, for additional sample collection. For 19 of the 23 IMD, biomarkers were detectable in blood (products) or urine. For the other four IMD, available biomarkers require invasive sampling such as: a lumbar puncture to collect CSF for proton-coupled folate transporter deficiency (*SLC46A1*, MIM #226050, *611672) and glucose transporter type 1 deficiency (*SLC2A1*, MIM #606777, #612126, #608885, #601042, *138140)), an intestinal biopsy or a breath test for congenital sucrase isomerase deficiency (*SI*, MIM #222900, *609845), and a skin biopsy for fibroblasts culturing for CAD trifunctional protein deficiency (*CAD*, MIM #616457, *114010).

### Combined results for search strategies

**Fig. 4:**
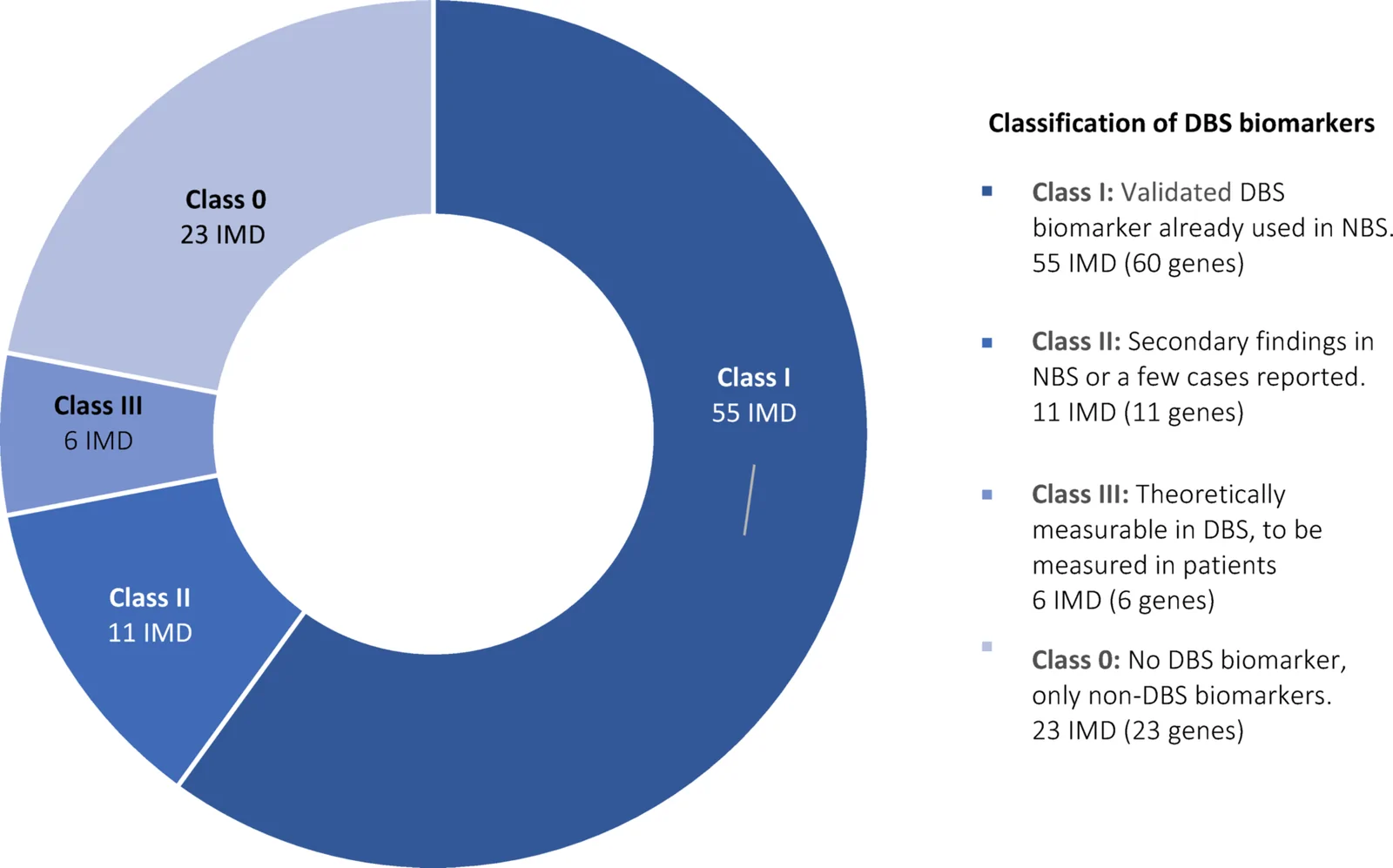
Classification of the DBS biomarkers identified for 100 genes associated with 95 IMD. IMD: inherited metabolic disorder DBS: dried blood spot

Overall, DBS biomarkers were identified for 72 out of 95 IMD, corresponding to 77 genes. 55 IMD (60 genes) with Class I biomarkers, including the 45 IMD (50 genes) from the CLIR database and 10 IMD (10 genes) from the systematic literature review. Eleven IMD were classified as having Class II biomarkers, and six IMD were classified as having Class III biomarkers. The distribution across these categories is illustrated in Fig. 4. See Online Appendix A for a comprehensive overview of all results.

## Discussion

This study presents a comprehensive overview of the biomarkers available (particularly in DBS) for 95 treatable IMD associated with 100 corresponding genes. We identified 66 candidate IMD (71 genes) for NBS with an established DBS biomarker (at Class I or II), theoretically enabling a combined genetic and biochemical test strategy using the primary DBS sample obtained for routine NBS. For 6 IMD (6 genes), theoretical DBS biomarkers are described in the literature, but they have not yet been validated in patient DBS samples (Class III). For the remaining 23 IMD (23 genes), we found at least one biomarker in non-DBS materials, requiring referral for additional sampling, either as part of the routine NBS process or diagnostic confirmatory process.

Based on our results, a combined genetic and biochemical strategy is potentially feasible for at least 66 IMD (71 genes) (Class I and Class II). Of these, biomarkers for 55 IMD (60/71 genes) are already incorporated in existing NBS programs. Class III are not yet ready for implementation in real-world NBS programs. For some of the disorders, the sensitivity and specificity of the biomarkers are unknown at NBS age. Their levels may not only depend on the well-known cofactors such as biological sex, birth weight, age at heel prick, gestational age, maternal diet or disease, and quality of the blood spot, but also by metabolic state, becoming abnormal only during a metabolic crisis. This is particularly relevant, for IMD affecting glucose metabolism, such as glucokinase deficiency (*GCK*, MIM #606176, #602485, #125853, #125851, *138079) and hepatic glycogen synthase deficiency (GSD0a) (*GYS2*, MIM #240600, *138571). For these IMD, biomarker levels are strongly influenced by the newborn’s fasting state, making the timing of sampling a critical factor for sensitivity. Genetic testing is not affected by these factors and may offer a solution. Careful decisions based on validation studies should therefore be made about whether to include these disorders in NBS and/or whether disorder-specific charts for referral and subsequent diagnostic testing should be made for each IMD to overcome the limitations of biochemical NBS tests.

Overall, genetic-based NBS holds great promise, and larger-scale studies are currently being performed to establish its feasibility and performance. However, NGS-NBS inherently shows lower sensitivity compared to traditional biochemical NBS programs (Adhikari et al., 2020; Bick et al., 2025; Chen et al., 2023; Kiewiet et al., 2024). For example, Bick et al. reported a sensitivity of 80.3% for 80 treatable IMD using NGS-techniques only, and estimated that this would lead to up to 650 patients with IMD from the Recommended Uniform Screening Panel being missed in the United States annually (Bick et al., 2025). The relatively low sensitivity they observe is in part explained by conservative variant interpretation criteria and by not reporting VUS. Including heterozygous P/LP variants with VUS (in AR disorders) would have reclassified 58 additional individuals as true positives, increasing the overall sensitivity to 89.4%. Other factors contributing to the reduced sensitivity they observed were incomplete penetrance, incomplete genotype─phenotype correlations, and disparities in variant interpretation due to ancestry bias in genomic reference databases. Well-known limitations of genomic databases like ClinVar (Landrum et al., 2020) and the Genome Aggregation Database (gnomAD) (Gudmundsson et al., 2022) are attributable to the fact that sequencing data is mostly obtained from non-diverse populations (Gudmundsson et al., 2022). A combined NBS strategy incorporating both genetic and biochemical testing may address these limitations. Biochemical tests can aid in the interpretation of variants (including VUS), thereby increasing the sensitivity of a genetics-based NBS. A combined strategy will also guide the interpretation of the two variants’ configuration in autosomal recessive (AR) genes, reducing the false-positive rate of genetic-NBS. This was reinforced by findings from Chen et al. (2023), where they found that *cis*-configuration occurred in 7/55 of all possible compound heterozygous cases (Chen et al., 2023). Long-read genome sequencing also offers a promising way to improve haplotype resolution, but its high cost and challenges for large-scale implementation remain significant hurdles.

NBS is already known to increase the observed prevalence of disorders by detecting children with mild, late-onset, or adult-onset phenotypes that would otherwise remain undiagnosed. However, disorders without clinical penetrance do not meet the W&J principles (J.M.G. Wilson, 1968; Schnabel-Besson et al., 2024), and late- or adult-onset forms are generally not considered suitable targets for NBS (Langeveld et al., 2025). There are examples of IMD in the current NBS where biochemical testing provides prognostic information on the phenotype and urgency of treatment. One example is very long chain acyl-CoA dehydrogenase deficiency (VLCADD) (*ACADVL*, MIM #201475, *609575), where DBS C18:2-carnitine could predict the clinical severity of the (Schwantje et al., 2026). Discriminating between mild- and severe presentations may avoid overdiagnosis of patients with a low risk of (Diekman et al., 2015). Similar ways of discriminating between mild- and severe forms of IMD are available for maple syrup urine disease (MSUD) (*BCKDHA/BCKDHB/DBT*, MIM #248600, #620699, *608348, *248611 (Stroek et al., 2020) or for medium-chain acyl-CoA dehydrogenase (MCAD) deficiency (*ACADM*, MIM #201450, *607008) (Jager et al., 2019). In addition, in clinical practice, the presence of biomarkers is not only used to support the pathogenicity of a genetic finding but also serves as an independent tool to decide on the initiation of treatment.” However, even established biomarkers, such as elevated concentrations of phenylalanine in PKU (*PAH*, MIM *612349, #261600), which are widely accepted as a clear indication to initiate treatment, do not invariably result in clinical diseases if remained undetected for some time (Van Vliet et al., 2018). We expect that such dilemmas will persist with the introduction of combined NBS strategies.

The potential advantage of biomarkers in predicting phenotypes is not uniformly applicable across all conditions. Genes that are associated with complex genotypes and/or unclear genotype–phenotype correlations, combined with poorly performing biomarkers, limit the suitability for inclusion in a combined NBS strategy. An example of a complex gene structure is *GBA*, which is affected by the highly homologous pseudogene *GBAP1*, complicating variant interpretation (Toffoli et al., 2022). In addition, *GBA-*associated Gaucher disease (*GBA*, MIM * 606463, #230800, #230900, #231000, #230105, #608013) exhibits a broad phenotypic spectrum ranging from asymptomatic to severe neuronopathic disease, with only limited ability of the DBS-based biomarkers (e.g. β-glucocerebrosidase activity and lyso-Gb1) to reliably predict disease severity. Therefore, even if Gaucher disease has a Class I biomarker, its implementation into NBS requires further evaluation.

IMD might be more common and less predictable in terms of penetrance and clinical severity than previously recognized, when evaluated using genetic data alone (Gold et al., 2025). Kiewiet et al. validated a variant filtering strategy for NGS-NBS using a background cohort of 4,833 presumed healthy parents to assess how many NBS positives would be reported based on NGS results (Kiewiet et al., 2024). They reviewed the same 95 IMD used in our study and found that three of the 4,833 individuals (0.06%) harbored a homozygous (L)P variant that would be reported in NBS, and four additional individuals would be added if a VUS in combination with an (L)P variant (in AR IMD) was considered. All individuals were associated with asymptomatic or mild phenotypes, representing potential false-positives. It is unknown if these mild phenotypes would have been picked up by the current biochemical NBS.

Approaches using AI may also help in reducing (potential) false-positives in NGS-NBS. Kingsmore *et al*. were able to reduce the number of “false-positive” NGS-results due to (mild) (L)P variants not associated with severe childhood disorders through federated training of an algorithm on genetic data from large healthy-population UK biobanks, a process called ‘purifying hyperselection’ (Kingsmore et al., 2024). Their approach reduced the number of (L)P variants by 293 (from 53,855 to 53,562) variants in 52 genes, including eight genes present in our gene list (*ACADVL*,* ALDOB*,* BTD*,* GALT*,* GCH1*,* IDUA*,* SLC22A5* and *SLC37A4)*. For eight genes, the number of false-positive diplotypes decreased by approximately 4.4% in the UK Biobank (Kingsmore et al., 2024). With phenotype-driven reporting, exclusion of mild forms, and integration of biochemical testing, the PPV may increase. Over time, it is expected that the phenotype prediction by genotype will also improve through the creation of international databases of genetic variants, biochemical data, and clinical phenotypes of children found positive in genetic NBS. To maximize benefits and minimize harm in this era of expanding NBS technology, careful selection and precise definition of targeted disorders remain essential.

Currently, practical considerations are still hampering implementation of a combined NBS, particularly, the difference in turn-around-times between biochemical and genetic tests. Biochemical test results will be available earlier than NGS results when using a parallel approach. Biochemical analysis is typically completed within 1–2 days after receipt of samples, whereas NGS in NBS setting commonly requires 5─7 days (Kiewiet et al., 2024). For some IMD, e.g., X-linked adrenoleukodystrophy (*ABCD1*, MIM #300100, *300371), a delay of several days is unlikely to affect outcomes. However, for disorders in which each hour of delay is critical, e.g., MCAD (*ACADM*, MIM #201450, *607008), immediate referral and initiation of treatment after a positive biochemical result may be the preferred course of action. Umbilical cord blood or immediate postpartum newborn blood sampling on a DBS card might offer a solution to shorten turn-around-times for time-critical NBS conditions. However, it has not been established whether the biomarkers used in NBS, alone or in different combinations, retain the same sensitivity when sampled immediately after birth. Cord blood sampling for NGS (which is not time-sensitive) and biochemical analysis in DBS taken in the regular NBS program, would enable earlier NGS results, while maintaining the same biochemical sensitivity and turn-around times. Long-read genomic sequencing is also expected to reasonably reduce turn-around-time (Smits et al., 2025). Alongside turn-around-times, costs are also expected to decrease in the foreseeable future, further supporting a combined approach that may become faster and cost-effective if the number of disorders included in NBS substantially expands.

Regardless of the NBS strategy, for every positive NBS result, an infant should be referred for definitive diagnostic procedures and follow-up. Some IMD need a functional test that reaches beyond the regular diagnostic testing in blood or urine. Well-designed confirmatory follow-up plans should be established before implementing either of the (NGS-) NBS strategies. In addition, the healthcare burden may shift depending on the design of a genetic-biochemical NBS strategy. We expect that a combined approach will reduce rather than increase the clinical burden, as the high number of false-positive test results in a biochemical-only approach is likely to decrease significantly. Furthermore, immediate biochemical confirmation of genetic variants will reduce the burden of communicating uncertain or less predictable genetic test results. We recognize that this is a paradigm shift in NBS that has not been tested at a large scale. Therefore, the impact of a combined NBS on healthcare systems should first be carefully evaluated in large-scale implementation studies.

A limitation of this study is the heterogeneity of biomarker evidence across IMD. While some IMD biomarkers are well described in the literature, others are reported only in isolated case reports, often due to disease rarity or intrinsic variability of the biomarker Consequently, it was not possible to reliably assess the sensitivity and specificity of all included biomarkers, as sufficient data on diagnostic performance were not available for each IMD. Second, several CLIR entries in Online Appendix A are based on very small patient numbers and drawing conclusions regarding sensitivity from such limited dataset should be done with caution. Third, although the systematic literature was performed according to the PRISMA guidelines (Page et al., 2021) (see Online Appendix C), no formal risk of bias assessment was performed. In addition, our assessment remains theoretical, but our findings support the development of a large-scale study to investigate a combined genetic-biochemical approach for these 95 IMD in practice. This will generate more substantiated data on the sensitivity and specificity of our biomarkers and a combined genetic and biochemical NBS strategy. Characteristics of different NGS (exome sequencing and (long-read) genome sequencing) and biochemical techniques (tandem mass-spectrometry, isoelectric focusing, enzyme analysis, metabolomics) should also be considered, but these technical specifications were not the focus of our study.

Rather than viewing genetic- and biochemical testing as competing strategies in NBS, their complementary strengths should be embraced and further explored. Together, genetic and biochemical testing can enhance NBS programs by increasing the NPV and PPV, improving the interpretation of variants, and improving prediction of disease severity, age of onset, and penetrance. In addition to genetic testing, application of other -omics methods in DBS, including (untargeted) metabolomics, lipidomics, and proteomics, is a promising route for the discovery of novel biomarkers or biomarker combinations for NBS-eligible diseases that currently have no DBS biomarker (Ashenden et al., 2024). The collaboration between the biochemical and genetic disciplines in NBS represents an opportunity that should be actively pursued.

## Supplementary Information

Below is the link to the electronic supplementary material.

Supplementary material 1 (DOCX 446.9 kb)

## Data Availability

All relevant data are contained within the manuscript and appendices. The boxplots from CLIR, search strings for individual IMD can be made available upon request to the corresponding author.
