# Supplementary material for: Next-generation newborn screening: feasibility of combined genetic and biochemical testing for 95 treatable inherited metabolic disorders
Source: Metabolomics. 2026 Jun 25;22(4):105. doi: 10.1007/s11306-026-02486-6 (PMC13303320; doi:10.1007/s11306-026-02486-6)
Supplement: Supplementary file 1 — Supplementary material 1 (DOCX 446.9 kb) [file 11306_2026_2486_MOESM1_ESM.docx]

**Appendix A.** A comprehensive overview of available biochemical tests for 95 IMD corresponding to 100 genes.

| **Inherited Metabolic Disorder** | **Associated gene** | **MIM**  **Pheno**  **type** | **MIM**  **Locus** | **Biomarkers or biochemical tests**  **in DBS** | **Classification** | **Biomarkers or biochemical tests**  **in non-DBS material*** | **Reported cases**  **(in DBS)** |
| --- | --- | --- | --- | --- | --- | --- | --- |
| 1. 3-hydroxy-3-methylglutaryl-CoA lyase deficiency | *HMGCL* | 246450 | 613898 | C5OH, C5OH(U) (CLIR) | 1 |  | 37 |
| 1. 3-Methylcrotonyl-CoA carboxylase 1 deficiency (synonym: 3-methylcrotonylglycinuria type 1) | *MCCC1* | 210200 | 609010 | C5OH, C5OH/Cit, C5OH/Gly, C5OH/Xle, C5OH/Val, C5OH/Ala, C5OH/Met, C5OH(U) (CLIR) | 1 |  | 551 |
| 1. 3-Methylcrotonyl-CoA carboxylase 2 deficiency (synonym: 3-methylcrotonylglycinuria type 2) | *MCCC2* | 210210 | 609014 | C5OH, C5OH/Cit, C5OH/Gly, C5OH/Xle, C5OH/Val, C5OH/Ala, C5OH/Met, C5OH(U) (CLIR) | 1 |  | 551 |
| 1. 3β-Hydroxy-Δ5-C27-steroid oxidoreductase deficiency | *HSD3B7* | [607765](http://omim.org/entry/607765) | 607764 | HSD3B7-BA1: glyco 3β,7α,12α-trihydroxy-5-cholenoic acid 3-sulfate (Muto et al., 2023),  HSD3B7-BA2: tauro 3β,7α,12α-trihydroxy-5-cholenoic acid 3-sulfate (Muto et al., 2023) | 2 | Urine (spots): BA profile (Ferreira et al., 2019; Mizuochi et al., 2010; Naritaka et al., 2019)  Plasma: Bilirubin, ALP, ASAT/ALAT, BA profile, 25-Hydroxy-Vitamin D, Vitamin E, ALP, ASAT/ALAT (Ferreira et al., 2019) BA profile (Ferreira et al., 2019; Mizuochi et al., 2010) | 2 |
| 1. 6-Pyruvoyl-tetrahydropterin synthase deficiency | *PTS* | 261640 | 612719 | Phe, Phe/Tyr, Met/Phe,  Val/Phe (CLIR) | 1 |  | 19 |
| 1. Adenosine deaminase 1 deficiency | *ADA* | 102700 | 608958 | 2-Deoxyadenosine, adenosine (Azzari et al., 2011; La Marca et al., 2014; Ombrone et al., 2016) | 1 | Erythrocytes: adenosine deaminase enzyme activity (Cagdas et al., 2018; La Marca et al., 2014)  Platelets: deoxyadenosine triphosphate (dATP) accumulation and ATP depletion (Simmonds et al., 1984)  Urine: adenosine and deoxyadenosine (Azzari et al., 2011) Plasma and serum: lymphocytopenia (Kohn et al., 2021) | 7 |
| 1. AKT2 superactivity (synonym: hypoinsulinemic hypoglycemia with hemihypertrophy) | *AKT2* | 240900 | 164731 |  | 0 | Blood and serum: hypoinsulinemic hypoglycemia with undetectable insulin (Arya et al., 2014; Dushar et al., 2021; Ferreira et al., 2019; Garg et al., 2015) | 0 |
| 1. Alanine-glyoxylate aminotransferase deficiency (synonym: Primary hyperoxaluria type 1) | *AGXT* | [259900](http://omim.org/entry/259900) | 604285 |  | 0 | Urine (spots): glycolate oxalate(Ferreira et al., 2019; Hoppe et al., 2025)  Plasma and serum: oxalate, glycolate(Ferreira et al., 2019; Hoppe et al., 2025) | 1 |
| 1. Aldolase B deficiency (synonym: hereditary fructose intolerance) | *ALDOB* | 229600 | 612724 | Possible option: transferrin glycosylation analysis in DBS (Bogdańska et al., 2021; Wada et al., 2022; Wolking et al., 2019) | 3 | Plasma: sialotransferrin type I profile (Evelina Maines et al., 2024)  Urine: reducing substances, negative glucose dipstick test (Betzler et al., 2024)  Liver or intestinal biopsy: aldolase activity (Betzler et al., 2024; Ferreira et al., 2019; James et al., 1996; Tolan, 1995) | 0 |
| 1. Amnionless deficiency (synonym: Imerslund-Gräsbeck disease, Norwegian type) | *AMN* | 618882 | 605799 | MMA (CLIR) | 1 |  | 169 |
| 1. Apolipoprotein C2 deficiency | *APOC2* | 207750 | 608083 | Lipid profile in DBS triglycerides (TG) comparable to plasma TG (Fallaize et al., 2016; Ferreira et al., 2023; Held et al., 2023) | 3 | Blood and serum: severe hypertriglyceridemia (Ferreira et al., 2019; Yoldas Celik et al., 2024)  Plasma and serum: lipoprotein lipase activity, cholesterol, TG (Ferreira et al., 2019) | 0 |
| 1. Apolipoprotein E deficiency (synonym: dysbetalipoproteinemia) | *APOE* | [617347](http://omim.org/entry/617347) | 107741 | Possible option: APOE (ɛ4) protein levels in DBS (Deza‐Lougovski et al., 2024)  Lipid profile (TC) in DBS (Fallaize et al., 2016; Ferreira et al., 2023; Held et al., 2023) | 3 | Plasma: ApoE protein distribution HDL/ VLDL (Elangovan et al., 2022)  Blood and serum: TC(Fallaize et al., 2016; Lehtimäki et al., 1994), LDL cholesterol and TG (Hellgren et al., 2015; Lehtimäki et al., 1994), ApoE protein levels (Hellgren et al., 2015),  ApoE phenotyping with IEF and immunoblotting (Ciomartan et al., 1999) | 0 |
| 1. Arginase deficiency (synonym: argininemia) | *ARG1* | 207800 | 608313 | Arg, Arg/Orn, Arg/Phe, Arg/Ala, Cit/Arg, C4/Arg, C3/Arg, C18:1/Arg (CLIR) | 1 |  | 30 |
| 1. Arginineglycine amidinotransferase (AGAT) deficiency | *GATM* | 612718,  134600 | 602360 | C8/Cre, C0/Cre, C5/Cre, Val/Cre, C12/Cre, Xle/Cre, C4/Cre, Arg/Cre, Tyr/Cre, Ala/Cre, Cit/Cre, Pro/Cre, C18:1/Cre, Phe/Cre, C16/Cre, C8/Cre, Met/Cre, C0/Crn, Cre/Gly, C3/Crn (CLIR) | 1 |  | 3 |
| 1. Argininosuccinate lyase deficiency | *ASL* | 207900 | 608310 | Asa, Asa/C18:1, Asa/C16, Asa/C3, C12/Asa (CLIR) | 1 |  | 120 |
| 1. Argininosuccinate synthetase deficiency (synonym: citrullinemia type 1) | *ASS1* | [215700](http://omim.org/entry/215700) | 603470 | Cit, Cit/Phe, Met/Cit, Pro/Cit, ACs/Cit, Glu/Cit, Gln/Cit, Ala/Cit (CLIR) | 1 |  | 217 |
| 1. Arylsulfatase A deficiency   (synonym: metachromatic leukodystrophy) | *ARSA* | 250100 | 607574 | Sulfatides (16:1-OH-sulfatide, 16:0-sulfatide), arylsulfatase A (Bekri et al., 2024; Saville et al., 2017) | 1 | After 1 month of age in CSF: protein (Ferreira et al., 2019)  After 1 month of age in urine: sulfatides(Ferreira et al., 2019) | 0 |
| 1. Autosomal recessive GTP cyclohydrolase 1 deficiency | G*CH1* | 233910, 128230 | 600225 | Phe, Phe/Tyr, Met/Phe,  Val/phe (CLIR) | 1 |  | 19 |
| 1. Bile acid-CoA:amino acid N-acyltransferase deficiency | *BAAT* | 619232 | 602938 | Possible option: Unconjugated BA in DBS (Muto et al., 2023), but no BAAT-patients have been reported | 3 | Serum: bile acid profile, almost completely unconjugated C24 bile acids (no glycine or taurine conjugates) (Hadžić et al., 2012; Nguyen et al., 2024; Setchell et al., 2013), calcium, phosphate (Nguyen et al., 2024)  Blood/Plasma: BA (no glycine or taurine conjugates), direct bilirubin, ALT, GGT (Ferreira et al., 2019; Nguyen et al., 2024; Setchell et al., 2013)  Urine: BA (no glycine or taurine conjugates) (Clayton, 2011; Setchell et al., 2013), cholanoids, bile alcohols (Clayton, 2011)  Blood: vitamin A, D and E (Ferreira et al., 2019; Setchell et al., 2013)  Liver biopsy: BAAT protein(Clayton, 2011; Hadžić et al., 2012; Setchell et al., 2013) | 0 |
| 1. Biotinidase deficiency | *BTD* | [253260](http://omim.org/entry/253260) | 609019 | BIOT IF, BIOT A, BIOT F,  BIOT C: BIOT F%, C5OH(C4DC)/BIOT A,  Suac/BIOT A, C12:1/BIOT A, C5DC(C6OH)/BIOT A, C5/BIOT A, C0/BIOT A, C2/BIOT A, C14:1/BIOT A, C18:1/BIOT A, Met/BIOT A, C14:2/BIOT A, C16:1/BIOT A, C8/BIOT A, C10/BIOT A, C4/BIOT A, C6/BIOT A, C18/BIOT A, C3DC(C4OH)/BIOT A, C12/BIOT A, C14/BIOT A, C10:1/BIOT A, C18:1OH/BIOT A, C14OH/BIOT A, C16OH/BIOT A, C8:1/BIOT A, C18OH/BIOT A, BIOT A/TRECs, BIOT A/Ala, BIOT A/Val, BIOT A/Xle, BIOT A/Pro, BIOT A/Gly, BIOT A/Phe, BIOT A/Orn (CLIR) | 1 |  | BIOT C: 126  BIOT P: 121 |
| 1. Branched-chain ketoacid dehydrogenase E1α deficiency (synonym: maple syrup urine disease type 1a, MSUD1a) | *BCKDHA* | 248600 | 608348 | Allo-Ile**,  Second-tier: Allo-Ile**/Val (CLIR) | 1 |  | 288 |
| 1. Branched-chain ketoacid dehydrogenase E1β deficiency (synonym: maple syrup urine disease type 1b, MSUD1b) | *BCKDHB* | 248600 | 248611 | Allo-Ile**,  Second-tier: Allo-Ile**/Val (CLIR) | 1 |  | 288 |
| 1. Branched-chain ketoacid dehydrogenase kinase deficiency | *BCKDK* | 614923 | 614901 | Xle,  Second-tier: Leu, Xle/Phe (CLIR) | 1 |  | 23 |
| 1. CAD trifunctional protein deficiency | *CAD* | 616457 | 114010 |  | 0 | Fibroblasts: pyrimidine nucleotides in CAD-deficient fibroblasts (Rymen et al., 2020); UDP, UDP-glucose, UDP-N-acetylglucosamine, CTP and UTP (Koch et al., 2017) | 0 |
| 1. Carbamoyl phosphate synthetase 1 deficiency | *CPS1* | [237300](http://omim.org/entry/237300) | 608307 | Cit (CLIR) | 1 |  | 71 |
| 1. Carbonic anhydrase VA deficiency | *CA5A* | [615751](http://omim.org/entry/615751) | 114761 |  | 0 | Liver biopsy: CA-VA activity (Van Karnebeek et al., 2014)  Plasma: ammonia, glucose, Gln, Ala, Pro, Cit and Arg (Van Karnebeek et al., 2014)  Urine: lactic acid, b-hydroxybutyric acid, acetoacetic acid, carboxylase substrates and related metabolites (3-methylcrotonylglycine, propionylglycine, 2-ketoglutarate, 3-hydroxyisovaleric acid, 3-hydroxypropionic acid), adipic acid, fumaric acid, sebacic acid, suberic acid, orotic acid (normal) (Ferreira et al., 2019; Van Karnebeek et al., 2014) | 0 |
| 1. Carnitine palmitoyltransferase 1A deficiency | *CPT1A* | [255120](http://omim.org/entry/255120) | 600528 | C0, C0/(C16+C18), C16:1OH  (Artic variant, no biomarkers) (CLIR) | 1 |  | 30 |
| 1. Carnitine palmitoyltransferase 2 deficiency | *CPT2* | 600649,  608836, 255110 | 600650 | (C16+C18:1)/C3, C16/C2, (C16+C18)/C2, C3/C16 (CLIR) | 1 |  | 69 |
| 1. Carnitine-acylcarnitine translocase deficiency | *SLC25A20* | 212138 | 613698 | (C16+C18:1)/C3, C16/C2, (C16+C18)/C2, C3/C16 (CLIR) | 1 |  | 69 |
| 1. Congenital sucrase-isomaltase deficiency | *SI* | 222900 | 609845 |  | 0 | Intestinal biopsy: sucrase isomaltase activity (Ferreira et al., 2019; Reinshagen et al., 2008)  Breath test: 13C-sucrose (Robayo-Torres et al., 2009) | 0 |
| 1. Copper-transporting ATPase β subunit deficiency (synonym: Wilson disease (WD)) | *ATP7B* | 277900 | 606882 | Proteomic-based multiplex assay to detect ATP7B peptide as a surrogate marker for the protein(Klippel et al., 2025),  Immuno-SRM's can detect ATP7B peptides on DBS (Collins et al., 2018; Jung et al., 2017; Poskanzer et al., 2020),  Caeruloplasmin (deWilde et al., 2008; Hahn, 2014; Ombrone et al., 2016) | 1 | Serum: low caeruloplasmin and copper (Ferreira et al., 2019; Hahn, 2014; Shribman et al., 2021)  Urine: copper (Ferreira et al., 2019; Shribman et al., 2021)  Liver biopsy: copper (Ferreira et al., 2019; Hahn, 2014; Shribman et al., 2021) | ? |
| 1. CTP synthase 1 deficiency | *CTPS1* | 615897 | 123860 |  | 0 | Lymphocytes: CTP synthase activity (Chinen et al., 2015; Martin et al., 2014) | 0 |
| 1. Cystathionine β-synthase deficiency (synonym: classic homocystinuria) | *CBS* | 236200 | 613381 | Hcy, Met (CLIR) | 1 |  | 83 |
| 1. Cystinosis | *CTNS* | 219800, 219900,  219750 | 606272 | Sedoheptulose (in patients with 57-kb deletion) (Wamelink et al., 2011)  Chitotriosidase enzyme activity on DBS (Elmonem et al., 2014; Veys et al., 2023) | 2/3 | Leukocytes and fibroblasts: cystine (Ferreira & Gahl, 2017; Ferreira et al., 2019; Gahl, 2003; Hohenfellner, Elenberg, et al., 2022; Hohenfellner, Nießl, et al., 2022; Hohenfellner et al., 2019; Nießl et al., 2022)  Plasma: chitotriosidase enzyme activity (Elmonem et al., 2014; Veys et al., 2023)  Urine: amino acids, phosphate(Ferreira et al., 2019) | 16 |
| 1. Dihydrolipoyl transacylase deficiency (synonyms: maple syrup urine disease type 2 (MSUD 2), branched-chain ketoacid dehydrogenase E2 deficiency) | *DBT* | 620699 | 248610 | Allo-Ile**,  Second-tier: Allo-Ile**/Val (CLIR) | 1 |  | 288 |
| 1. Dihydropteridine reductase deficiency | *QDPR* | 248600 | 612676 | Phe, Phe/Tyr, Phe/(C3+C16), Met/Phe, Val/Phe, Cit/Phe, Xle/Phe (CLIR) | 1 |  | 5 |
| 1. DNAJC12 deficiency | *DNAJC12* | 617384 | 606060 | Phe(Anikster et al., 2017; Blau et al., 2018; Chen et al., 2022; Deng et al., 2024; Donnelly et al., 2024; Feng et al., 2019; Fino et al., 2023; Gunes & Senturk, 2023; M. Li et al., 2020; Martín-Rivada et al., 2022; Navarrete et al., 2019; Tendi et al., 2023; Wang et al., 2023; Wong et al., 2023; Wong et al., 2024)  Pterins, DHPR activity (Anikster et al., 2017) | 2 | Urine: DHPR, and pterins (neopterin, biopterin, and primapterin) (Anikster et al., 2017; Wong et al., 2023)  CSF: biogenic amines (5-hydroxyindoleacetic acid, homovanillic acid) and pterines (Blau et al., 2018; Ferreira et al., 2019)  Plasma: Phe (Ferreira et al., 2019) |  |
| 1. Electron transfer flavoprotein dehydrogenase deficiency (synonym: glutaric acidemia type 2C, multiple acyl-CoA dehydrogenase deficiency type 2C (MADD type 2C)) | *ETFDH* | 231680 | 231675 | 2OHG (CLIR) | 1 |  | 99 |
| 1. Electron transfer flavoprotein α subunit deficiency (synonym: glutaric acidemia type 2A, multiple acyl-CoA dehydrogenase deficiency type 2A   (MADD type 2A)) | *ETFA* | 231680 | 608053 | 2OHG (CLIR) | 1 |  | 99 |
| 1. Electron transfer flavoprotein β subunit deficiency (synonym: glutaric acidemia type 2B, multiple acyl-CoA dehydrogenase deficiency type 2B   (MADD type 2B)) | *ETFB* | 231680 | 130410 | 2OHG (CLIR) | 1 |  | 99 |
| 1. Ferroportin deficiency   (synonym: hereditary hemochromatosis type 4) | *SLC40A1* | 606069 | 604653 |  | 0 | Serum: ferritin saturation, transferrin saturation (never tested in newborns) (Badar et al., 2016; Chen et al., 2015; Ferreira et al., 2019; Schimanski et al., 2005; Sham et al., 2005; Wu et al., 2021; Zarifian Yeganeh et al., 2024), hepcidin-25 (Hattori et al., 2012; Kaneko et al., 2010)  Liver biopsy: iron deposition (Badar et al., 2016; Chen et al., 2015; Sham et al., 2005; Wu et al., 2021) | 0 |
| 1. Folate receptor α deficiency   (synonym: neurodegeneration due to cerebral folate transport deficiency) | *FOLR1* | 613068 | 136430 |  | 0 | Serum: 5-MTHF (Ohba et al., 2013), folate receptor alpha autoantibody (Frye & Rossignol, 2012)  CSF: 5-MTHF (Dreha-Kulaczewski et al., 2024; Ferreira et al., 2019; Frye & Rossignol, 2012; Ohba et al., 2013; Wilcken, 2012)  Magnetic Resonance Spectroscopy (MRS): choline, inositol (Ferreira et al., 2019) | 0 |
| 1. Fructose-1,6-bisphosphatase deficiency | *FBP1* | [229700](http://omim.org/entry/229700) | 611570 |  | 0 | Plasma and serum: glucose, Ala, ketones, acidosis, phosphate, uric acid, TG (pseudo hypertriglyceridemia) (Ferreira et al., 2019; Kuhara, 2001; Mayatepek et al., 2010; Ni et al., 2024; Pinto et al., 2018)  Urine: lactate, ketones, glycerol and glycerol-3-phosphate (most specific for FBP1), uric acid (Ferreira et al., 2019; Kuhara, 2001)  Liver biopsy and leucocytes: fructose-1,6-bisphosphatase activity (Moon et al., 2011) | 0 |
| 1. Fumarylacetoacetase deficiency (synonym: Tyrosinemia type 1) | *FAH* | 276700 | 613871 | Suac, Suac(U),  Suac(U)/Ala, Suac(U)/Phe, Suac(U)/Gln(Lys) Suac(U)/C2, Suac(U)/C3, Suac(U)/Orn, Suac(U)/Xle, Suac(U)/C18, Suac(U)/C16, Suac(U)/C18:1, Suac(U)/C0, C5/Suac(U), C14/Suac(U), C4/Suac(U), C12:1/Suac(U), C14:1/Suac(U), C6/Suac(U), C5OH(U)/Suac(U), C5DC(U)/Suac(U), C8/Suac(U), C12/Suac(U), C10:1/Suac(U), C18:1OH/Suac(U), C3DC(C4OH)/Suac(U), C10/Suac(U), C18OH/Suac(U), C16OH/Suac(U), C8:1/Suac(U) (CLIR) | 1 |  | 73 |
| 1. Galactokinase deficiency | *GALK1* | 230200 | 604313 | Secondary finding in NBS for classic galactosemia: TGAL (galactose + galactose-1-phosphate), galactose, GALK activity (Hennermann et al., 2011; Kikuchi et al., 2021; Li et al., 2011; Maroulis et al., 2023; Park et al., 2009; F. Porta et al., 2015; Reich et al., 2002; Schulpis et al., 2017; Stroek et al., 2018) | 1 | Erythrocytes and fibroblasts: GALK activity (Janzen et al., 2011; Kalaydjieva et al., 1999; Pasquali et al., 2018),  Urine: galactitol (Rubio-Gozalbo et al., 2021)  Plasma: galactose (Ferreira et al., 2019) | 39 |
| 1. Galactose-1-phosphate uridylyltransferase deficiency   (synonym: classic galactosemia) | *GALT* | 230400 | 606999 | GALT (Classic variant):  GALT, C8/GALT, Met/GALT, Xle/GALT, C3/GALT, C14:1/GALT, Val/GALT, C2/GALT, Pro/GALT, Phe/GALT, C18:1/GALT, C18/GALT, Orn/GALT, C4/GALT, C12/GALT, C0/GALT, C5/GALT, Cit/GALT, Tyr/GALT, C16/GALT,  BIOT A/GALT, GALT/Ala, GALT/Gly (CLIR)  GALT (Duarte variant):  GALT, Xle/GALT, Orn/GALT, Val/GALT, Pro/GALT, C0/GALT, Phe/GALT, Tyr/GALT, Met/GALT, Cit/GALT, BIOT A/GALT, C8/GALT, C18/GALT (CLIR) | 1 |  | GALT GG: 60  GALT DG: 158 |
| 1. Glucocerebrosidase deficiency   (synonym: Gaucher disease type I, II, III, IIIC) | *GBA* | 230800, 230900, 231000, 230105,  608013 | 606463 | GBA activity (Kang et al., 2017; Wolf et al., 2018),  Lysosphingolipids including: LysoGb1(Burlina et al., 2019; Cozma et al., 2020; Dinur et al., 2023; Dinur et al., 2022; Dubiela et al., 2024; Dubiela et al., 2023; Gary et al., 2018; Hurvitz et al., 2019; Kishnani et al., 2022; Mak & Cowan, 2021; Malinová et al., 2023; Pawliński et al., 2020; Polo et al., 2019; Revel-Vilk et al., 2020; Saville et al., 2020; Savolainen et al., 2021; Tang et al., 2021; Van Baelen et al., 2023; Vernet Machado Bressan Wilke et al., 2024; Zimran et al., 2025), Lyso-SM, LysoGb3, lyso-dihexosylceramide, glucosylceramide isoforms (for example: C16:0) (Di Rocco et al., 2023; Dinur et al., 2023; Ji et al., 2015; Meikle et al., 2004; Polo et al., 2019; Spiewak et al., 2023; Zimran et al., 2025),  GAGs, α-galactosidase A (Gragnaniello et al., 2023), acid β-glucocerebrosidase (Di Rocco et al., 2023)  chitotriosidase (Pacheco & Uribe, 2013; Woo et al., 2014) | 1 | Blood, plasma and fibroblasts: LysoGb1, acid β-glucosidase activity, chitotriosidase (Burlina et al., 2019; Ferreira et al., 2019; Giuffrida et al., 2023; Gragnaniello et al., 2022; Malinová et al., 2023; Woo et al., 2014; Zhang et al., 2017) Serum: tartrate-resistant acid phosphatase (Malinová et al., 2023) angiotensin converting enzyme (Pacheco & Uribe, 2013)  glucosylsphingosine (Ferreira et al., 2019)  CSF: LysoGb1 (Gragnaniello et al., 2023; Revel-Vilk et al., 2020; Vernet Machado Bressan Wilke et al., 2024) |  |
| 1. Glucokinase deficiency | *GCK* | 606176,  602485,  125853,  125851 | 138079 | Blood glucose (highly variable, not sensitive or specific) (McDonald et al., 2017; Shepherd et al., 2020)) | 0 | Plasma and serum: glucose, free fatty acids, ketones during hypoglycemia, insulin (Ferreira et al., 2019)  Urine: ketones during hypoglycemia (Ferreira et al., 2019) | 0 |
| 1. Glucose transporter 2 deficiency (synonym: Fanconi-Bickel syndrome,   glycogen storage disease XI) | *SLC2A2* | [227810](http://omim.org/entry/227810) | 138160 | Secondary finding in NBS for classic galactosemia: TGAL (Gal + Gal-1-P) (Grünert et al., 2021; Müller et al., 1997; Peduto et al., 2004; Francesco Porta et al., 2015; Yoo et al., 2002) | 2 | Blood, plasma and serum: galactose (Ferreira et al., 2019; Grünert et al., 2021; Yoo et al., 2002),  fasting glucose, fed glucose, ALP, BIOT, (Molares-Vila et al., 2021; Paesold-Burda et al., 2007)  ASAT, ALAT, uric acid, phosphate, TC, TG (Ferreira et al., 2019)  Urine: glucosuria, mild proteinuria (Grünert et al., 2021; Yoo et al., 2002), hyperaminoaciduria, galactitol, galactose, calcium, glucose, phosphate (Ferreira et al., 2019), tetraglucoside (Overduin et al., 2025)  Liver biopsy: glycogen accumulation (Ferreira et al., 2019; Müller et al., 1997) |  |
| 1. Glucose-6-phosphatase deficiency (synonym: glycogen storage disease type 1a) | *G6PC* | 232200 | 613742 |  | 0 | Blood, plasma and serum: glucose, lactic acid, uric acid, ASAT, ALAT, fasting ketones, BIOT, carnitine profile, TG, TC, carnitine profile (Bindi et al., 2021; Ferreira et al., 2019; Groen et al., 2025; Paesold-Burda et al., 2007)  Urine: fasting ketones, fasting lactate, uric acid (Ferreira et al., 2019), tetraglucoside (Manwaring et al., 2012)  Liver biopsy: glucose-6-phosphatase activity, glycogen (Ferreira et al., 2019) | 0 |
| 1. Glucose-6-phosphate transporter deficiency (synonym: glycogen storage disease type 1b) | *SLC37A4* | 232220, 232240,  619525 | 602671 | 1,5-anhydroglucitol (not measured in newborns yet) (Dewulf et al., 2023) | 2 | Plasma: 1,5-anhydroglucitol (Dewulf et al., 2023),  lactic acid, liver enzymes uric acid, BIOT, fasting ketones, TC, TG (Betzler et al., 2024; Bindi et al., 2021; Ferreira et al., 2019)  Liver biopsy: pale-staining, swollen hepatocytes, steatosis, and nuclear hyperglycogenation, glucose-6-phosphatase (Betzler et al., 2024; Ferreira et al., 2019)  Blood: neutrophil count (Ferreira et al., 2019)  Urine: fasting ketones, fasting lactate, uric acid (Ferreira et al., 2019) |  |
| 1. Glucose Transporter type 1 deficiency   (GLUT 1 deficiency syndrome (GLUT1DS)) | *SLC2A1* | 606777,  612126,  608885,  601042 | 138140 |  | 0 | CSF or blood glucose ratio after 4-6 hour fasting <0.4-0.45 (Brockmann, 2011; Falsaperla et al., 2021; Ferreira et al., 2019; Galosi et al., 2020; Mauri et al., 2022; Vulturar et al., 2022)  CSF: glucose (Galosi et al., 2020); lactate (Betzler et al., 2024; Campistol & Plecko, 2015; Ferreira et al., 2019; Kwon, 2018),  After 1 month of age in CSF: galactonic acid, gluconic acid, xylose-α1-3-glucose, xylose-α1-3-xylose-α1-3-glucose (Ferreira et al., 2019),  Blood or erythrocytes: glucose, lactate (Betzler et al., 2024; Falsaperla et al., 2021; Kwon, 2018), GLUT1 enzyme activity (Ferreira et al., 2019) | 0 |
| 1. Glutamate dehydrogenase superactivity (synonym: hyperinsulinism-hyperammonemia syndrome) | *GLUD1* | 606762 | 138130 |  | 0 | Serum and plasma: ammonia, non-ketogenic hypoglycemia + excessive increase in serum insulin or detectable plasma C-peptide, free fatty acids (Ferrara et al., 2016; Ferreira et al., 2019; Xu et al., 2019; Zeng & Sang, 2023)  Urine: ketones, alpha-ketoglutarate (Ferreira et al., 2019) | 0 |
| 1. Glutaryl-CoA dehydrogenase deficiency (synonym: glutaric acidemia type 1) | *GCDH* | 231670 | 608801 | GLUT, C5DC(U)/Ala, C5DC(D) (CLIR) | 1 |  | 281 |
| 1. Glycogen debranching enzyme deficiency (synonyms: glycogen storage disease type 3 (GSD3), Cori-Forbes disease, limit dextrinosis) | *AGL* | [232400](http://omim.org/entry/232400) | 610860 |  | 0 | Blood: fasting glucose, lipids, ketones (Urtizberea et al., 2023)  Urine: tetraglucoside (Manwaring et al., 2012; Overduin et al., 2025) fasting ketones, fasting lactate, uric acid (Ferreira et al., 2019)  Liver biopsy: glycogen, amylo-1,6-glucosidase activity (Ferreira et al., 2019; Santer et al., 2001)  Plasma and serum: ASAT, ALAT, BIOT, creatine kinase, fasting ketones, fasting glucose, fastinglactate, uric acid, TC, TG (Ferreira et al., 2019)  Leucocytes: amylo-1,6-glucosidase (Ferreira et al., 2019) |  |
| 1. Glycosylphosphatidylinositol-anchored high-density lipoprotein-binding protein 1 (GPIHBP1) deficiency | *GPIHBP1* | 615947 | 612757 | Lipid profiles in DBS, triglycerides comparable to plasma TG (Fallaize et al., 2016; Ferreira et al., 2023; Held et al., 2023) | 3 | Blood: GPIHBP1 protein quantification (ELISA) (Hu et al., 2017; Miyashita et al., 2018)  Serum: TG (Ferreira et al., 2019; Guay et al., 2013; Iacocca et al., 2019; Kurooka et al., 2023; Schaefer et al., 2019), fasting cholesterol, HDL-cholesterol, direct LDL-C, and lipoprotein a (Schaefer et al., 2019) |  |
| 1. Guanidinoacetate methyltransferase deficiency (GAMT) | *GAMT* | 612736 | 601240 | Guac, Guac/Arg, Guac/Orn, Guac/Cre, Guac/Cit, Guac/Ala, Guac/Pro, Guac/C2, Guac/Phe, Guac/C0, Guac/Met, Guac/Gly, Guac/C18:1, Guac/Crn, Guac/Tyr, Guac/Xle, Guac/Val (CLIR) | 1 |  | 6 |
| 1. Hemojuvelin deficiency   (synonym: juvenile hemochromatosis) | *HJV* | 602390 | 608374 |  | 0 | Blood and serum: hepcidin-25(Kaneko et al., 2010)  hepcidin, hemoglobin, ferritin, transferrin saturation (Al-Rawaf et al., 2023; Badar et al., 2016; Ferreira et al., 2019; Roetto et al., 2000)  Liver biopsy: iron (normal) (Ferreira et al., 2019) |  |
| 1. Hepatic glycogen synthase deficiency (synonym: glycogen storage disease type 0a (GSD0a)) | *GYS2* | [240600](http://omim.org/entry/240600) | 138571 |  | 0 | Blood: fasting ketotic hypoglycemia (Arko et al., 2020; Kasapkara Ç et al., 2017; Matei et al., 2019; Miwa et al., 2010; Nessa et al., 2012; Soggia et al., 2010; Tagliaferri et al., 2022), Ala, lactate(Arko et al., 2020; Kasapkara Ç et al., 2017; Tagliaferri et al., 2022)  Plasma: (low) fasting mannose (Miwa et al., 2010), fasting ketones, glucose, lactate(Ferreira et al., 2019)  Urine: fasting ketones (Ferreira et al., 2019)  Liver biopsy: hepatic glycogen (Arko et al., 2020; Bachrach et al., 2002; Ferreira et al., 2019; Orho et al., 1998; Spiegel et al., 2007; Weinstein et al., 2006), glycogen synthase activity (Ferreira et al., 2019; Orho et al., 1998) |  |
| 1. Holocarboxylase synthetase deficiency | *HLCS* | 253270 | 609018 | C5OH, C5OH(U) (CLIR) | 1 |  | 16 |
| 1. Homocystinuria, cblDv1 type | *MMADHC* | [277410](http://omim.org/entry/277410),  620953,  620952 | 611935 | Hcy, Met (CLIR) | 1 |  | 83 |
| 1. Intestinal sodium-glucose cotransporter 1 deficiency (synonym: glucose-galactose malabsorption (GGM)) | *SLC5A1* | [606824](http://omim.org/entry/606824) | 182380 |  | 0 | Stool: galactose, glucose (Atay et al., 2017), positive reducing substance/sugar (Alamoudi et al., 2021; Ferreira et al., 2019; Kasahara et al., 2001; Katz et al., 2023), acidic stool (Kasahara et al., 2001)  Small bowel biopsy: normal (Alruwaili & Alshdayed, 2023; Vallaeys et al., 2013; Wang et al., 2020)  Functional tests: glucose and galactose uptake by enterocytes, fructose loading test (oral), galactose loading test (oral) + glucose loading test (oral) (Ferreira et al., 2019) |  |
| 1. Isovaleryl-CoA dehydrogenase deficiency (synonym: isovaleric academia (IVA)) | *IVD* | 243500 | 607036 | C5, C5/C3, C5/C2, C5/C8, C5/C0 (CLIR) | 1 |  | 217 |
| 1. Lipoprotein lipase deficiency | *LPL* | 238600,  144250 | 609708 | No test available, but: false positive NBS results (for BIOT deficiency) or contaminated DBS samples because of severe hyperchylomicronemia and extreme hypertriglyceridemia (Ashraf et al., 2017; Santer et al., 2005)  Possible option: lipid profiles (TG) in DBS (Fallaize et al., 2016; Ferreira et al., 2023; Held et al., 2023) | 3 | Blood or plasma: Viscous pink blood ("Pepto-Bismol like") due to extreme hypertriglyceridemia (Ashraf et al., 2017; Ferreira et al., 2019; Santer et al., 2005); TC(Ferreira et al., 2019);  Enzyme testing: lipoprotein lipase activity (Ferreira et al., 2019) | 0 |
| 1. Medium-chain acyl-CoA dehydrogenase deficiency (MCAD) | *ACADM* | [201450](http://omim.org/entry/201450) | 607008 | C8, C6, C8/Gly, C8/C18:1, C8/C16, C8/Ala, C8/Val, C8/C2, C8/C0, C8/C4DC, C8/C8:1, C6/Phe, C3DC(D)/C8, C4/C8, C5OH(D)/C8, C12/C8, C5/C8, C5DC(U)/C8, C16OH/C8, C5OH(U)/C8, C14:1/C8, C4OH/C8 (CLIR) | 1 |  | 1990 |
| 1. Methylmalonic acidemia and homocystinuria, cblC type | *MMACHC* | [277400](http://omim.org/entry/277400) | 609831 | MMA (CLIR) | 1 |  | 169 |
| 1. Methylmalonic acidemia due to methylmalonyl-CoA epimerase deficiency | *MCEE* | [251120](http://omim.org/entry/251120) | 608419 | C3 (E. Maines et al., 2024; Manoli et al., 1993), MCA, MMA (normal), Hcy (normal) (E. Maines et al., 2024) | 2 | Blood: MMA (Manoli et al., 1993), C3 (Fernández-Lainez et al., 2024)  Urine: MMA (E. Maines et al., 2024; Manoli et al., 1993), 3-OH-isovaleric acid, MCA, 3-hydroxypropionic acid, propionyl glycine (Fernández-Lainez et al., 2024; Ferreira et al., 2019)  Enzymatic testing: methylmalonyl-CoA epimerase activity (Manoli et al., 1993) |  |
| 1. Methylmalonic acidemia due to methylmalonyl-CoA mutase deficiency | *MMUT* | 251000 | 609058 | MMA (CLIR) | 1 |  | 217 |
| 1. Methylmalonic acidemia, cblA type | *MMAA* | [251100](http://omim.org/entry/251100) | 607481 | MMA (CLIR) | 1 |  | 217 |
| 1. Mitochondrial 3-hydroxy-3-methylglutaryl-CoA synthase deficiency   (synonym: HMG-CoA synthase deficiency) | *HMGCS2* | 605911 | 600234 | C2/C0 (Decru et al., 2025), not sensitive in DBS | 2 | Urine: 4HMP (Decru et al., 2025)  Plasma: C2/C0 carnitine ratio (Decru et al., 2025) |  |
| 1. Mitochondrial acetoacetyl-CoA thiolase deficiency (synonym: beta-ketothiolase deficiency, T2) | *ACAT1* | [203750](http://omim.org/entry/203750) | 607809 | C4OH, C5OH (CLIR) | 1 |  | 36 |
| 1. Mitochondrial ornithine transporter deficiency (synonym: hyperornithinemia-hyperammonemia-homocitrullinuria syndrome) | *SLC25A15* | 238970 | 603861 | Orn (but only after a few days) (Auray-Blais et al., 2021; Fernando et al., 2021; La Marca et al., 2014; Sokoro et al., 2010) | 1 | Blood: Orn, ammonia (Ferreira et al., 2019; Wild et al., 2019)  Plasma: Orn, Gln, Cit, Ala, Arg, Met (Ferreira et al., 2019; Lee et al., 2014; Wild et al., 2019)  Urine: orotic acid (Auray-Blais et al., 2021; D'Apolito et al., 2010; Ferreira et al., 2019; Lee et al., 2014), uracil (Auray-Blais et al., 2021; Lee et al., 2014), homocitrulline (Al-Dirbashi et al., 2006; Ferreira et al., 2019; Lee et al., 2014; Wild et al., 2019), Crn (Al-Dirbashi et al., 2006)  Functional testing fibroblasts: 14C-ornithine incorporation (Ferreira et al., 2019)  Specific tests: (low) Factor VII, Factor X (Ferreira et al., 2019) |  |
| 1. N-acetylglutamate synthase deficiency | *NAGS* | 237310 | 608300 | Cit (CLIR) | 1 |  | 3 |
| 1. Ornithine aminotransferase deficiency (synonym: gyrate atrophy of choroid and retina) | *OAT* | 258870 | 613349 | Cit (CLIR) | 1 |  | 2 |
| 1. Ornithine transcarbamylase deficiency | *OTC* | 311250 | 300461 | Cit (CLIR) | 1 |  | 71 |
| 1. Phenylalanine hydroxylase deficiency (synonym: phenylketonuria (PKU)) | *PAH* | 261600 | 612349 | Phe, Phe/Tyr, Phe/(C3+C16), Val/Phe, Cit/Phe, Met/Phe, C18:1/Phe, Xle/Phe, (Phe) (CLIR) | 1 |  | 1565 |
| 1. Phosphoglucomutase 1 deficiency (PGM1-CDG) | *PGM1* | 614921 | 171900 | PGM1 enzymatic activity (Conte et al., 2020; Nolting et al., 2017; Wolking et al., 2019), Transferrin glycosylation analysis (Wolking et al., 2019) | 2 | Leucocytes: PGM1 enzymatic activity (Nolting et al., 2017)  Erythrocytes: enzyme activity (Gahr & Schröter, 1981)  Blood: ammonia, insulin during hypoglycemia (Ferreira et al., 2019)  Serum: (sialo)transferrin profile (Ferreira et al., 2019; Wada & Okamoto, 2022; Wolking et al., 2019), free fatty acids during hypoglycemia, ketones during hypoglycemia (Ferreira et al., 2019)  Plasma: creatine kinase, transaminases, glucose, antithrombin III (Ferreira et al., 2019)  Urine: ketones during hypoglycemia (Ferreira et al., 2019) |  |
| 1. Primary carnitine deficiency | *SLC22A5* | [212140](http://omim.org/entry/212140) | 603377 | C0 (CLIR) | 1 |  | 274 |
| 1. Propionic acidemia (PA) due to propionyl-CoA carboxylase α subunit deficiency | *PCCA* | 606054 | 232000 | MCA, C3, C3/C2, (C16+C18:1)/C3 (CLIR) | 1 |  | 160 |
| 1. Propionic acidemia (PA) due to propionyl-CoA carboxylase β subunit deficiency | *PCCB* | 606054 | 232050 | MCA, C3, C3/C2, (C16+C18:1)/C3 (CLIR) | 1 |  | 160 |
| 1. Proton-coupled folate transporter deficiency (synonym: hereditary folate malabsorption (HFM)) | *SLC46A1* | 229050 | 611672 |  | 0 | CSF: 5-MTHF / folate  Serum: folate (Borzutzky et al., 2009; Goldman, 1993; Gowda et al., 2021; Huddar et al., 2021; Kishimoto et al., 2014; Shin et al., 2011; Tan et al., 2017; Torres et al., 2015; Wang et al., 2015; Zhao et al., 2007)  Blood: pan-hypogammaglobulinemia (Kishimoto et al., 2014)  Transformed lymphocytes from blood: analysis of 5-MTHF transport (Zhao et al., 2007) |  |
| 1. Pterin-4-α-carbinolamine dehydratase deficiency | *PCBD1* | 264070 | 126090 | Phe, Phe/Tyr, Phe/(C3+C16), Met/Phe, Val/Phe, Cit/Phe, Xle/Phe (CLIR) | 1 |  | 5 |
| 1. Purine nucleoside phosphorylase deficiency | *PNP* | 613179 | 164050 | First-tier: deoxyguanosine, deoxyinosine  Second-tier: guanosine, inosine (La Marca et al., 2014; la Marca et al., 2016; Ombrone et al., 2016) | 1 | Blood and erythrocytes: uric acid (Shakerian et al., 2021), purine nucleoside phosphorylase enzyme activity (Ferreira et al., 2019)  Serum: inosine, T and/or B lymphopenia (Roifman et al., 2012; Shakerian et al., 2021)  CSF: Inosine, uric acid (Haijes et al., 2019)  Urine and plasma: uric acid, deoxyguanosine, deoxyinosine (Ferreira et al., 2019) |  |
| 1. Riboflavin transporter 2 deficiency (synonym: Brown-Vialetto-van Laere syndrome type 1) | *SLC52A3* | 211500,  211530 | 613350 | Biochemical profile of multiple acyl-CoA dehydrogenase deficiency (MADD) (Tummolo et al., 2022), but not sensitive  DBS: C4-C18 carnitine, free carnitines (Ferreira et al., 2019) | 2 | All biochemical tests are normal (Carreau et al., 2020)  Blood: flavins (Ferreira et al., 2019)  Plasma: C4-C18 carnitine, free carnitines(Ferreira et al., 2019)  Urine: ethylmalonic acid, glutaric acid, adipic acid, C6-C10 dicarboxylic acids, sebacic acid, suberic acid (Ferreira et al., 2019) |  |
| 1. Riboflavin transporter 3 deficiency (synonym: Brown-Vialetto-van Laere syndrome type 2) | *SLC52A2* | [614707](https://omim.org/entry/614707) | 607882 | Biochemical profile of multiple acyl-CoA dehydrogenase deficiency (MADD) (Tummolo et al., 2022), but not sensitive  DBS: C4-C18 carnitine, free carnitines (Ferreira et al., 2019) | 2 | All biochemical tests are normal (Carreau et al., 2020)  Blood: flavins (Ferreira et al., 2019)  Plasma: C4-C18 carnitine, free carnitines(Ferreira et al., 2019)  Urine: ethylmalonic acid, glutaric acid, adipic acid, C6-C10 dicarboxylic acids, sebacic acid, suberic acid (Ferreira et al., 2019) |  |
| 1. S-adenosylhomocysteine hydrolase deficiency | *AHCY* | 613752 | 180960 | Met, Met/Pro, Met/Phe (CLIR) | 1 |  | 106 |
| 1. Sterol 27-hydroxylase deficiency | *CYP27A1* | [213700](http://omim.org/entry/213700) | 606530 | First-tier GlcA-tetrol, GlcA-tetrol/t-CDCA  Second-tier: GlcA-tetrol, t-CDCA, t-THCA  combining those a 100% sensitivity can be achieved (Hong et al., 2020; Shao et al., 2024; Vaz et al., 2017; Vaz et al., 2023)  Other DBS biomarkers: 7α12α-C4 (Hong et al., 2020; Shao et al., 2024), t-THCA/GlcA-tetrol (Hong et al., 2020) | 1 | Blood: 7α12αC4, GlcA-tetrol (Shao et al., 2024)  Plasma: Cholestanol (Ferreira et al., 2019)  Urine: Cholestane pentol glucuronide (Ferreira et al., 2019)  Fibroblast enzyme testing: 27-Hydroxylase activity (Ferreira et al., 2019) |  |
| 1. Succinyl-CoA:3-oxoacid-CoA transferase deficiency (SCOT deficiency) | *OXCT1* | 245050 | 601424 | ACs are normal, mild or non-specific (Fukao et al., 2014; Hori et al., 2015) | 0 | Blood: total ketone bodies (Fukao et al., 2014)  Plasma: ketones, glucose (Ferreira et al., 2019)  Urine: ketone positive, 3-hydroxy-n-butyric acid, acetoacetate (Fukao et al., 2014), C6-C10 dicarboxylic acids (Ferreira et al., 2019)  Enzyme analysis and fibroblasts: SCOT-enzyme activity (Fukao et al., 2014; Schwade et al., 2017) |  |
| 1. Thiamine pyrophosphokinase deficiency | *TPK1* | [614458](http://omim.org/entry/614458) | 606370 |  | 0 | Blood and muscle: TPP concentration (Mascarenhas et al., 2024; Mayr et al., 2011; D. Zhao et al., 2023; Zhu et al., 2020)  Urine: 2-ketoglutaric acid (only in half of patients abnormal) (Banka et al., 2014; D. Zhao et al., 2023), α-ketoglutarate (D. Li et al., 2020; Mascarenhas et al., 2024; Zhu et al., 2020)  Plasma: thiamine (Mascarenhas et al., 2024), TPP (Schubert Baldo & Vilarinho, 2020)  Serum: lactate (Mascarenhas et al., 2024; Mayr et al., 2011; Zhu et al., 2020)  CSF: lactate can be normal (Li et al., 2022; Mascarenhas et al., 2024) |  |
| 1. Thiamine transporter 2 deficiency (synonym: biotin-thiamine-responsive basal ganglia disease) | *SLC19A3* | [607483](http://omim.org/entry/607483) | 606152 |  | 0 | CSF: pyruvate, lactate (Alfadhel et al., 2019), free thiamine (Schubert Baldo & Vilarinho, 2020)  Serum: amino acids (Alfadhel et al., 2019)  Urine: amino acids (Alfadhel et al., 2019), lactate (Ygberg et al., 2016) |  |
| 1. Transcobalamin II deficiency | *TCN2* | 275350 | 613441 | C3 has 2% overlap with the reference population***. No biomarkers with 0% overlap. (CLIR) | 1 |  | 12 |
| 1. Trifunctional protein α subunit deficiency (Synonym: long chain 3-hydroxyacyl-CoA dehydrogenase deficiency (LCHAD)) | *HADHA* | 609015,  609016 | 600890 | C16OH/C2, C16OH (CLIR) | 1 |  | 134 |
| 1. Tyrosine aminotransferase deficiency (synonyms: tyrosinemia type 2; Richner-Hanhart syndrome (RHS)) | *TAT* | 276600 | 613018 | Tyr, Tyr/Pro, (Xle+Val)/(Phe+Tyr), Phe/Tyr, Met/Tyr, C18:1/Tyr, Xle/Tyr (CLIR) | 1 |  | 16 |
| 1. Tyrosine hydroxylase deficiency | *TH* | 605407 | 191290 | No biomarkers in DBS (Wassenberg et al., 2021) | 0 | Serum: prolactin (12 out of 14 cases) (Wassenberg et al., 2021)  Urine: dopamine (Ferreira et al., 2019)  CSF: homovanillic acid (Bijarnia-Mahay et al., 2020; Wassenberg et al., 2021), 3-methoxy-4-hydroxyphenylglycol (MHPG) (Bijarnia-Mahay et al., 2020; Ferreira et al., 2019) |  |
| 1. Very long-chain acyl-CoA dehydrogenase deficiency (VLCAD) | *ACADVL* | [201475](http://omim.org/entry/201475) | 609575 | C14:1/Ala, C14:1/Gly, C14:1/C2, C14:1/Phe, C14:1/C16 (CLIR) | 1 |  | 504 |
| 1. X-linked adrenoleukodystrophy | *ABCD1* | 300100 | 300371 | Used in NBS 100% sensitive: C26:0-LysoPC (Baker et al., 2022; Billington et al., 2025; Haynes & De Jesús, 2015, 2016; Hong et al., 2018; Kettwig et al., 2021; Kilgore et al., 2023; Mashima et al., 2016; Morales-Romero et al., 2024; Natarajan et al., 2018; Ombrone et al., 2016; Tang et al., 2024; Teber et al., 2022; Videbæk et al., 2023)  C26:0-carnitine (less sensitive (83%) than C26:0-LysoPC) (Huffnagel et al., 2017; Van De Beek et al., 2016)  Other biomarkers: C26:0/C20:0, C26/C22:0, C24:0/C20:0 and C24:0/C22:0-LysoPC  (sensitivity: between 80% and 70% (Natarajan et al., 2018)), C20:0 LysoPC and C24:0 LysoPC (Wu et al., 2017)  Diagnostic biomarkers in older children:  C26:0-LysoPC, C24:0 and C26:0 (Tian et al., 2020) | 1 | Plasma: VLCFA accumulation(Billington et al., 2025; Ferreira et al., 2019; Ombrone et al., 2016), hexacosanoic acid 26:0 (Bonilla Guerrero et al., 2008; Kemp et al., 2016; Ombrone et al., 2016), C26:0/C22:0 and C24:0/C22:0 ratios (diagnostic biomarkers) (Huffnagel et al., 2019; Morales-Romero et al., 2024)  Tissues: VLCFA accumulation (Billington et al., 2025; Ferreira et al., 2019; Van De Beek et al., 2016) |  |
| 1. α-aminoadipic semialdehyde dehydrogenase deficiency (synonym: pyridoxine-dependent epilepsy) | *ALDH7A1* | 266100 | 107323 | 2-OPP (Damiano, Della Bona, Procopio, Guerrini, et al., 2025; Engelke et al., 2021; Pauly et al., 2025), 6-oxo-PIP (Coughlin et al., 2022; Damiano, Della Bona, Procopio, Guerrini, et al., 2025; Engelke et al., 2021; Pauly et al., 2025; Wempe et al., 2019), α-AASA, P6C (Jung et al., 2013; Mathew et al., 2018; Xue et al., 2019), PA (Mathew et al., 2018; Xue et al., 2019), α-AAA (Xue et al., 2019) | 1 | Blood: 6-oxo-PIP (Wempe et al., 2019), 2-OPP (Engelke et al., 2021), α-AASA, PA, P6C (Judy et al., 2024)  Plasma: 6-oxo-PIP (Damiano, Della Bona, Procopio, Gasperini, et al., 2025; Wempe et al., 2019), 2-OPP (Damiano, Della Bona, Procopio, Gasperini, et al., 2025), AASA, PA, P6C, α-AAA (Xue et al., 2019), two sets of diastereomeric derivatives of P6C (van Outersterp et al., 2021)  Serum: AASA, PA, P6C, α-AAA (Xue et al., 2019)  Urine (spots) or CSF: 6-oxo-PIP (Damiano, Della Bona, Procopio, Gasperini, et al., 2025; Wempe et al., 2019), 2-OPP, α-AASA, α-AAA, PA, P6C (Damiano, Della Bona, Procopio, Gasperini, et al., 2025; Judy et al., 2024; Xue et al., 2019), two sets of diastereomeric derivatives of P6C (van Outersterp et al., 2021) |  |
| 1. α-iduronidase deficiency (synonym: mucopolysaccharidosis type 1 (MPS1), Hurler syndrome, Scheie syndrome) | *IDUA* | 607014,  607015,  607016 | 252800 | DS, HS, IDUA (CLIR) | 1 |  | 56 |
| 1. α-tocopherol transfer protein deficiency (synonym: ataxia with isolated vitamin E deficiency) | *TTPA* | 277460 | 600415 |  | 0 | Plasma: Vitamin E (Elkamil et al., 2015; Jeridi et al., 2023; Manor & Morley, 2007)  Serum: Vitamin E (Zhang et al., 2022) |  |
| 1. Δ4-3-oxosteroid 5β-reductase deficiency (synonym: AKR1D1 deficiency; SRD5B1-congenital bile acid synthesis defect type 2) | *AKR1D1* | 235555 | 604741 | SRD5B1-BA1: glyco 7α,12α-dihydroxy-3-oxo-4-cholenoic acid,  SRD5B1-BA2: tauro 7α,12α-dihydroxy-3-oxo-4-cholenoic acid (Muto et al., 2023) | 2 | Urine: 3-oxo-Δ4 bile acids, Absence or abnormally low level of primary bile acids (Gardin et al., 2023; Gonzales et al., 2004; Yanagi et al., 2015; Zhang et al., 2019; J. Zhao et al., 2023), 7a,12a-dihydroxy-3-oxo-4-cholenoic acids (Clayton, 2011; Ferreira et al., 2019)  Serum: γ-glutamyltransferase, TBA (Gardin et al., 2023; Kimura et al., 2023), ALAT, conjugated bilirubin (Kimura et al., 2023),  Plasma: allochenodeoxycholic acid, allocholic acid, ASAT, ALAT, ALP, GGT, conjugated bilirubin (Ferreira et al., 2019) |  |

CLIR = Collaborative Laboratory Integrated Reports, DBS = Dried blood spot, IMD = Inherited metabolic disorder, NBS = Newborn screening.

Class 1: biomarkers or biochemical tests already used by one of the NBS programs. Class 2:  biomarkers or biochemical tests as either a secondary finding in a NBS program, or only a few cases reported in DBS. Class 3: biomarkers or biochemical tests measurable in DBS but never reported in patients with the specific IMD. Grade 0: No biomarker in DBS was found for this IMD. *An extensive literature for non-DBS biomarkers was only performed in no DBS biomarkers could be identified; therefore, this column is not intended to be exhaustive for every IMD. **The CLIR-reported ‘Allo-ile (allo-isoleucine)’ likely represents a calculated surrogate within the total leucine (Xle) signal (i.e. allo-Xle), as allo-isoleucine cannot be measured separately by flow injection tandem mass spectometry. ****TCN2* shows a small (≈2%) overlap with the reference population but retains its CLIR Class I classification based on established newborn screening practice and CLIR database annotation.

Abbreviations of Appendix A in alphabetical order:

ACs = Acylcarnitines, ADA = Adenosine deaminase, Ala = Alanine, Allo-Ile = Allo-isoleucine, ALP = Alkaline phosphatase, ALAT = Alanine Aminotransferase, Arg = Arginine, Asa = Argininosuccinic acid, ASAT = Aspartate Aminotransferase, ATP = Adenosine Triphosphate, BA = Bile acids, BIOT = Biotinidase, CA-VA = Carbonic anhydrase VA, Cit = Citrulline, , Cre = Creatine, Crn = Creatinine, CSF = Cerebrospinal fluid, CTP = Cytidine Triphosphate, C0 = Free carnitine, C2 = C2-carnitine or acetylcarnitine, C3 = Propionylcarnitine, C3DC = Malonylcarnitine, C4 = C4-carnitine or butyrylcarnitine/isobutyrylcarnitine, C4DC = methylmalonylcarnitine/succinylcarnitine, C4OH = 3-Hydroxybutyrylcarnitine, C5 = C5-carnitine or isovalerylcarnitine/2-methylbutyrylcarnitine, C5DC = Glutarylcarnitine, C5OH = 3-hydroxyisovalerylcarnitine, C6 = Hexanoylcarnitine, C6OH = 3-Hydroxyhexanoylcarnitine, C8 = Octanoylcarnitine, C8:1 = Octenoylcarnitine, C10 = Decanoylcarnitine, C10:1 = Decenoylcarnitine, C12 = Dodecanoylcarnitine, C12:1 = Dodecenoylcarnitine, C14 = Tetradecanoylcarnitine, C14OH = 3-Hydroxytetradecanoylcarnitine, C14:1 = Tetradecenoylcarnitine, C14:2 = Tetradecadienoylcarnitine, C16 = C16-carnitine, C16OH = 3-Hydroxypalmitoylcarnitine, C16:1 = Hexadecenoylcarnitine, C18 = C18-carnitine or stearoylcarnitine, C18OH = 18-Hydroxystearoylcarnitine, C18:1 = Oleoylcarnitine, C18:1OH = 3-Hydroxyoleoylcarnitine, C20:0 = C20-carnitine or arachidic acid, C22:0 = C22-carnitine, C24:0 = C24-carnitine or tetracosanoyl-carnitine, C26:0 = C26:0-carnitine or hexacosanoic acid, C26:0-LysoPC = C26:0-lysophosphatidylcholine, DS = Dermatan Sulfate, DHPR = Dihydropteridine Reductase, ELISA = Enzyme-Linked Immunosorbent Assay, GAGs = Glycosaminoglycans, GALK = Galactokinase, GALT = Galactose-1-phosphate Uridyltransferase, GBA = glucocerebrosidase, GGT = Gamma-glutamyltransferase or gamma-glutamyl transpeptidase, Gal = Galactose, Gln = Glutamine, GlcCer = Glucosylceramide, Glu = Glutamic acid , GLUT1 = Glucose Transporter type 1, Gly = Glycine, GlcA-tetrol = 5β-cholestane-3α,7α,12α,25-tetrol glucuronide, Guac = Guanidinoacetic acid, Hcy = Total homocysteine, HDL = High-density lipoprotein,, HS = Heparan Sulfate, IDUA =  Alpha-L-Iduronidase  (enzyme activity), IEF = Isoelectric focusing, LDL = Low-density lipoprotein, Leu = Leucine, Lys = Lysine, Lyso-Gb1 = Glucosylsphingosine, LysoPC = lysophosphatidylcholine, Lyso-SM = Lysosphingomyelin, MADD = Multiple acyl-CoA dehydrogenase deficiency, MCA = Methylcitric acid, Met = Methionine, MHPG = 3-methoxy-4-hydroxyphenylglycol, MMA = Methylmalonic acid, Orn = Ornithine, PA = Pipecolic acid, PGM1 = Phosphoglucomutase 1, Phe = Phenylalanine, Pro = Proline, P6C = Piperideine-6-carboxylate, SCOT = Succinyl-CoA: 3-oxoacid-CoA transferase, Suac = Succinylacetone, TBA = Total bile acids, TC = Total cholesterol, TG = Triglycerides, TGAL = galactose + galactose-1-phosphate, TPP = Thiamine pyrophosphate diphosphate, TRECs = T-cell Receptor Excision Circles, Tyr = Tyrosine, t-CDCA = Tauro-chenodeoxycholic acid, t-THCA = Tauro-trihydroxycholestanoic acid, U = Underivatized, UDP = Uridine Diphosphate, UTP = Uridine Triphosphate, Val = Valine, VLCFA = Very long-chain fatty acids, VLDL = Very low-density lipoprotein, Xle = Total leucine (Ile, Leu, Allo-Ile), 2OHG = 2-Hydroxyglutaric acid, 2-OPP = 2S,6S-/2S,6R-oxopropylpiperidine-2-carboxylic acid, 4HMP = 4-hydroxy-6-methyl-2-pyrone, , 5-MTHF = 5-methyltetrahydrofolate, 6-oxoPIP = 6-oxopiperidine-2-carboxylic acid, 7α12α-C4 = 7α,12α-dihydroxy-4-cholesten-3-one, α-AAA  = α-aminoadipic acid, α-AASA = α-aminoadipic semialdehyde.

**References**

1. Muto Y, Suzuki M, Takei H, et al. Dried blood spot-based newborn screening for bile acid synthesis disorders, Zellweger spectrum disorder, and Niemann-Pick type C1 by detection of bile acid metabolites. Article. *Molecular Genetics and Metabolism*. 2023;140(1-2)doi:10.1016/j.ymgme.2023.107703

2. Naritaka N, Suzuki M, Takei H, et al. Use of dried urine spots for screening of inborn errors of bile acid synthesis. *Pediatrics International*. 2019;61(5):489. doi:10.1111/ped.13852

3. Mizuochi T, Kimura A, Ueki I, et al. Molecular Genetic and Bile Acid Profiles in Two Japanese Patients With 3β-Hydroxy-Δ5-C27-Steroid Dehydrogenase/Isomerase Deficiency. *Pediatric Research*. 2010;68(3):258-263. doi:10.1203/pdr.0b013e3181eb0188

4. Ferreira CR, Van Karnebeek CDM, Vockley J, Blau N. A proposed nosology of inborn errors of metabolism. *Genetics in Medicine*. 2019;21(1):102-106. doi:10.1038/s41436-018-0022-8

5. La Marca G, Canessa C, Giocaliere E, et al. Diagnosis of immunodeficiency caused by a purine nucleoside phosphorylase defect by using tandem mass spectrometry on dried blood spots. *Journal of Allergy and Clinical Immunology*. 2014;134(1):155-159.e3. doi:10.1016/j.jaci.2014.01.040

6. Ombrone D, Giocaliere E, Forni G, Malvagia S, La Marca G. Expanded newborn screening by mass spectrometry: New tests, future perspectives. *Mass Spectrometry Reviews*. 2016;35(1):71-84. doi:10.1002/mas.21463

7. Azzari C, La Marca G, Resti M. Neonatal screening for severe combined immunodeficiency caused by an adenosine deaminase defect: A reliable and inexpensive method using tandem mass spectrometry. *Journal of Allergy and Clinical Immunology*. 2011;127(6):1394-1399. doi:10.1016/j.jaci.2011.03.040

8. Cagdas D, Gur Cetinkaya P, Karaatmaca B, et al. ADA Deficiency: Evaluation of the Clinical and Laboratory Features and the Outcome. *Journal of Clinical Immunology*. 2018/05/01 2018;38(4):484-493. doi:10.1007/s10875-018-0496-9

9. Simmonds HA, Goday A, Morris GS, Fairbanks LD, Levinsky RJ. dATP accumulation and ATP depletion in platelets in adenosine deaminase deficiency: significance for the immune response? *Biosci Rep*. Oct 1984;4(10):809-18. doi:10.1007/bf01138162

10. Kohn DB, Booth C, Shaw KL, et al. Autologous Ex Vivo Lentiviral Gene Therapy for Adenosine Deaminase Deficiency. *New England Journal of Medicine*. 2021;384(21):2002-2013. doi:10.1056/nejmoa2027675

11. Arya VB, Flanagan SE, Schober E, Rami-Merhar B, Ellard S, Hussain K. Activating <i>AKT2</i> Mutation: Hypoinsulinemic Hypoketotic Hypoglycemia. *The Journal of Clinical Endocrinology & Metabolism*. 2014;99(2):391-394. doi:10.1210/jc.2013-3228

12. Garg N, Bademci G, Foster J, 2nd, Sıklar Z, Berberoglu M, Tekin M. MORFAN Syndrome: An Infantile Hypoinsulinemic Hypoketotic Hypoglycemia Due to an AKT2 Mutation. *J Pediatr*. Aug 2015;167(2):489-91. doi:10.1016/j.jpeds.2015.04.069

13. Dushar M, Nowaczyk J, Pyrżak B, et al. Efficacy and safety of sirolimus therapy in familial hypoinsulinemic hypoglycemia caused by AKT2 mutation inherited from the mosaic father. *Eur J Med Genet*. Dec 2021;64(12):104368. doi:10.1016/j.ejmg.2021.104368

14. Hoppe B, Martin-Higueras C, Borghese L, et al. Effective Newborn Screening for Type 1 and 3 Primary Hyperoxaluria. *Kidney Int Rep*. Jan 2025;10(1):177-183. doi:10.1016/j.ekir.2024.10.006

15. Wolking AB, Park JH, Grüneberg M, et al. Transferrin glycosylation analysis from dried blood spot cards and capillary blood samples. *J Chromatogr B Analyt Technol Biomed Life Sci*. Feb 1 2019;1106-1107:64-70. doi:10.1016/j.jchromb.2019.01.004

16. Bogdańska A, Kozłowski D, Pajdowska M, Lipiński P, Tylki-Szymańska A. Transferrin isoform analysis from dried blood spots and serum samples by gel isoelectric focusing for screening congenital disorders of glycosylation. *Acta Biochim Pol*. Mar 5 2021;68(1):139-142. doi:10.18388/abp.2020_5576

17. Wada Y, Kadoya M, Okamoto N. Mass Spectrometry of Transferrin and Apolipoprotein CIII from Dried Blood Spots for Congenital Disorders of Glycosylation. *Mass Spectrom (Tokyo)*. 2022;11(1):A0113. doi:10.5702/massspectrometry.A0113

18. Maines E, Gugelmo G, Maiorana A, et al. The role of the analysis of sialotransferrin isoforms in the management of hereditary fructose intolerance: a systematic review. *Journal of Diabetes & Metabolic Disorders*. 2024;24(1)doi:10.1007/s40200-024-01527-y

19. Betzler IR, Hempel M, Mütze U, et al. Comparative analysis of gene and disease selection in genomic newborn screening studies. *Journal of Inherited Metabolic Disease*. 2024;47(5):945-970. doi:10.1002/jimd.12750

20. Tolan DR. Molecular basis of hereditary fructose intolerance: mutations and polymorphisms in the human aldolase B gene. *Hum Mutat*. 1995;6(3):210-8. doi:10.1002/humu.1380060303

21. James CL, Rellos P, Ali M, Heeley AF, Cox TM. Neonatal screening for hereditary fructose intolerance: frequency of the most common mutant aldolase B allele (A149P) in the British population. *Journal of Medical Genetics*. 1996;33(10):837-841. doi:10.1136/jmg.33.10.837

22. Fallaize R, Celis-Morales C, Macready AL, et al. The effect of the apolipoprotein E genotype on response to personalized dietary advice intervention: findings from the Food4Me randomized controlled trial. *Am J Clin Nutr*. Sep 2016;104(3):827-36. doi:10.3945/ajcn.116.135012

23. Ferreira HB, Melo T, Rocha H, Paiva A, Domingues P, Domingues MR. Lipid profile variability in children at different ages measured in dried blood spots. *Mol Omics*. Mar 27 2023;19(3):229-237. doi:10.1039/d2mo00206j

24. Held PK, Lasarev M, Zhang X, et al. Familial Hypercholesterolemia Biomarker Distribution in Dried Blood Spots. *J Pediatr*. Aug 2023;259:113469. doi:10.1016/j.jpeds.2023.113469

25. Yoldas Celik M, Canda E, Yazici H, et al. Long-term clinical outcomes and management of hypertriglyceridemia in children with Apo-CII deficiency. *Nutr Metab Cardiovasc Dis*. Jul 2024;34(7):1798-1806. doi:10.1016/j.numecd.2024.02.006

26. Deza‐Lougovski YI, Weiss LM, Horton HM, et al. Circulating apoE4 protein levels from dried blood spots predict cognitive function in a large population‐based survey setting. *Alzheimer's & Dementia*. 2024;20(11):7613-7623. doi:10.1002/alz.14224

27. Elangovan B, N TR, Subrahmanian M. Apolipoprotein-E Gene Polymorphism and Lipid Composition among IUGR and AGA Neonates. *J Pediatr Genet*. Sep 2022;11(3):179-184. doi:10.1055/s-0040-1722212

28. Lehtimäki T, Porkka K, Viikari J, Ehnholm C, Akerblom HK, Nikkari T. Apolipoprotein E phenotypes and serum lipids in newborns and 3-year-old children: the Cardiovascular Risk in Young Finns Study. *Pediatrics*. Oct 1994;94(4 Pt 1):489-93.

29. Hellgren G, Engström E, Smith LE, Löfqvist C, Hellström A. Effect of Preterm Birth on Postnatal Apolipoprotein and Adipocytokine Profiles. *Neonatology*. 2015;108(1):16-22. doi:10.1159/000381278

30. Ciomartan T, Uchiyama TA, Makoto. Serum lipoproteins and apolipoprotein E in infants with congenital hypothyroidism. *Pediatrics International*. 1999;41(3):249-252. doi:10.1046/j.1442-200x.1999.01063.x

31. Saville JT, Smith NJ, Fletcher JM, Fuller M. Quantification of plasma sulfatides by mass spectrometry: Utility for metachromatic leukodystrophy. *Anal Chim Acta*. Feb 22 2017;955:79-85. doi:10.1016/j.aca.2016.12.002

32. Bekri S, Bley A, Brown HA, et al. Higher precision, first tier newborn screening for metachromatic leukodystrophy using 16:1-OH-sulfatide. *Mol Genet Metab*. May 2024;142(1):108436. doi:10.1016/j.ymgme.2024.108436

33. Hadžić N, Bull LN, Clayton PT, Knisely AS. Diagnosis in bile acid-CoA: amino acid N-acyltransferase deficiency. *World J Gastroenterol*. Jul 7 2012;18(25):3322-6. doi:10.3748/wjg.v18.i25.3322

34. Setchell KD, Heubi JE, Shah S, et al. Genetic defects in bile acid conjugation cause fat-soluble vitamin deficiency. *Gastroenterology*. May 2013;144(5):945-955.e6; quiz e14-5. doi:10.1053/j.gastro.2013.02.004

35. Nguyen DQ, Can TBN, Vu CD, et al. Identification of a novel BAAT frameshift mutation in a female child diagnosed with skeletal dysplasia: A case report. *Medicine (Baltimore)*. Sep 6 2024;103(36):e39509. doi:10.1097/md.0000000000039509

36. Clayton PT. Disorders of bile acid synthesis. *J Inherit Metab Dis*. Jun 2011;34(3):593-604. doi:10.1007/s10545-010-9259-3

37. Rymen D, Lindhout M, Spanou M, et al. Expanding the clinical and genetic spectrum of CAD deficiency: an epileptic encephalopathy treatable with uridine supplementation. *Genet Med*. Oct 2020;22(10):1589-1597. doi:10.1038/s41436-020-0933-z

38. Koch J, Mayr JA, Alhaddad B, et al. CAD mutations and uridine-responsive epileptic encephalopathy. *Brain*. Feb 2017;140(2):279-286. doi:10.1093/brain/aww300

39. Van Karnebeek CDM, Shevell M, Zschocke J, Moeschler JB, Stockler S. The metabolic evaluation of the child with an intellectual developmental disorder: Diagnostic algorithm for identification of treatable causes and new digital resource. *Molecular Genetics and Metabolism*. 2014;111(4):428-438. doi:10.1016/j.ymgme.2014.01.011

40. Reinshagen K, Keller KM, Haase B, Leeb T, Naim HY, Zimmer KP. Mosaic pattern of sucrase isomaltase deficiency in two brothers. *Pediatr Res*. Jan 2008;63(1):79-83. doi:10.1203/PDR.0b013e31815b4bac

41. Robayo-Torres CC, Opekun AR, Quezada-Calvillo R, et al. 13C-breath tests for sucrose digestion in congenital sucrase isomaltase-deficient and sacrosidase-supplemented patients. *J Pediatr Gastroenterol Nutr*. Apr 2009;48(4):412-8. doi:10.1097/mpg.0b013e318180cd09

42. Klippel C, Park J, Sandin S, et al. Advancing Newborn Screening in Washington State: A Novel Multiplexed LC-MS/MS Proteomic Assay for Wilson Disease and Inborn Errors of Immunity. *International Journal of Neonatal Screening*. 2025;11(1):6. doi:10.3390/ijns11010006

43. Poskanzer SA, Thies J, Collins CJ, et al. The co-occurrence of Wilson disease and X-linked agammaglobulinemia in one family highlights the promising diagnostic potential of proteolytic analysis. *Mol Genet Genomic Med*. Apr 2020;8(4):e1172. doi:10.1002/mgg3.1172

44. Collins CJ, Chang IJ, Jung S, et al. Rapid Multiplexed Proteomic Screening for Primary Immunodeficiency Disorders From Dried Blood Spots. *Front Immunol*. 2018;9:2756. doi:10.3389/fimmu.2018.02756

45. Jung S, Whiteaker JR, Zhao L, Yoo H-W, Paulovich AG, Hahn SH. Quantification of ATP7B Protein in Dried Blood Spots by Peptide Immuno-SRM as a Potential Screen for Wilson’s Disease. *Journal of Proteome Research*. 2017;16(2):862-871. doi:10.1021/acs.jproteome.6b00828

46. Hahn SH. Population screening for Wilson's disease. *Ann N Y Acad Sci*. May 2014;1315:64-9. doi:10.1111/nyas.12423

47. deWilde A, Sadilkova K, Sadilek M, Vasta V, Hahn SH. Tryptic peptide analysis of ceruloplasmin in dried blood spots using liquid chromatography-tandem mass spectrometry: application to newborn screening. *Clin Chem*. Dec 2008;54(12):1961-8. doi:10.1373/clinchem.2008.111989

48. Shribman S, Poujois A, Bandmann O, Czlonkowska A, Warner TT. Wilson's disease: update on pathogenesis, biomarkers and treatments. *J Neurol Neurosurg Psychiatry*. Oct 2021;92(10):1053-1061. doi:10.1136/jnnp-2021-326123

49. Martin E, Palmic N, Sanquer S, et al. CTP synthase 1 deficiency in humans reveals its central role in lymphocyte proliferation. *Nature*. 2014;510(7504):288-292. doi:10.1038/nature13386

50. Chinen J, Notarangelo LD, Shearer WT. Advances in basic and clinical immunology in 2014. *J Allergy Clin Immunol*. May 2015;135(5):1132-41. doi:10.1016/j.jaci.2015.02.037

51. Wamelink MM, Struys EA, Jansen EE, et al. Elevated concentrations of sedoheptulose in bloodspots of patients with cystinosis caused by the 57-kb deletion: implications for diagnostics and neonatal screening. *Mol Genet Metab*. Mar 2011;102(3):339-42. doi:10.1016/j.ymgme.2010.12.002

52. Veys K, Zadora W, Hohenfellner K, et al. Outcome of infantile nephropathic cystinosis depends on early intervention, not genotype: A multicenter sibling cohort study. *Journal of Inherited Metabolic Disease*. 2023;46(1):43-54. doi:10.1002/jimd.12562

53. Elmonem MA, Makar SH, van den Heuvel L, et al. Clinical utility of chitotriosidase enzyme activity in nephropathic cystinosis. *Orphanet J Rare Dis*. Nov 19 2014;9:155. doi:10.1186/s13023-014-0155-z

54. Hohenfellner K, Elenberg E, Ariceta G, Nesterova G, Soliman NA, Topaloglu R. Newborn Screening: Review of its Impact for Cystinosis. *Cells*. Mar 25 2022;11(7)doi:10.3390/cells11071109

55. Hohenfellner K, Nießl C, Haffner D, et al. Beneficial effects of starting oral cysteamine treatment in the first 2 months of life on glomerular and tubular kidney function in infantile nephropathic cystinosis. *Mol Genet Metab*. Aug 2022;136(4):282-288. doi:10.1016/j.ymgme.2022.06.009

56. Hohenfellner K, Rauch F, Ariceta G, et al. Management of bone disease in cystinosis: Statement from an international conference. *Journal of Inherited Metabolic Disease*. 2019;42(5):1019-1029. doi:10.1002/jimd.12134

57. Ferreira CR, Gahl WA. Lysosomal storage diseases. *Transl Sci Rare Dis*. May 25 2017;2(1-2):1-71. doi:10.3233/trd-160005

58. Gahl WA. Early oral cysteamine therapy for nephropathic cystinosis. *Eur J Pediatr*. Dec 2003;162 Suppl 1:S38-41. doi:10.1007/s00431-003-1349-x

59. Nießl C, Boulesteix AL, Oh J, et al. Relationship between age at initiation of cysteamine treatment, adherence with therapy, and glomerular kidney function in infantile nephropathic cystinosis. *Mol Genet Metab*. Aug 2022;136(4):268-273. doi:10.1016/j.ymgme.2022.06.010

60. Donnelly C, Estrella L, Ginevic I, Ganesh J. A Case of DNAJC12-Deficient Hyperphenylalaninemia Detected on Newborn Screening: Clinical Outcomes from Early Detection. *Int J Neonatal Screen*. Jan 17 2024;10(1)doi:10.3390/ijns10010007

61. Deng IB, Follett J, Bu M, Farrer MJ. DNAJC12 in Monoamine Metabolism, Neurodevelopment, and Neurodegeneration. *Mov Disord*. Feb 2024;39(2):249-258. doi:10.1002/mds.29677

62. Wong TS, Wong SSN, Kwok AMK, et al. DNAJC12 Deficiency, an Emerging Condition Picked Up by Newborn Screening: A Case Illustration and a Novel Variant Identified. *Int J Neonatal Screen*. Nov 19 2024;10(4)doi:10.3390/ijns10040074

63. Chen A, Pan Y, Chen J. Clinical, genetic, and experimental research of hyperphenylalaninemia. *Front Genet*. 2022;13:1051153. doi:10.3389/fgene.2022.1051153

64. Fino E, Barbato A, Scaturro GM, Procopio E, Balestrini S. DNAJC12 deficiency: Mild hyperphenylalaninemia and neurological impairment in two siblings. *Mol Genet Metab Rep*. Dec 2023;37:101008. doi:10.1016/j.ymgmr.2023.101008

65. Wong RSH, Mohammad S, Parayil Sankaran B, et al. Developmental delay and non-phenylketonuria (PKU) hyperphenylalaninemia in DNAJC12 deficiency: Case and approach. *Brain Dev*. Oct 2023;45(9):523-531. doi:10.1016/j.braindev.2023.04.004

66. Wang L, Ma D, Sun Y, et al. Identification of two novel DNAJC12 gene variants in a patient with mild hyperphenylalaninemia. *Gene*. Jun 15 2023;869:147397. doi:10.1016/j.gene.2023.147397

67. Gunes D, Senturk L. A rare cause of hyperphenylalaninemia: four cases from a single family with DNAJC12 deficiency. *J Pediatr Endocrinol Metab*. Aug 28 2023;36(8):791-797. doi:10.1515/jpem-2023-0049

68. Tendi EA, Morello G, Guarnaccia M, et al. Detection of Single-Nucleotide and Copy Number Defects Underlying Hyperphenylalaninemia by Next-Generation Sequencing. *Biomedicines*. Jul 4 2023;11(7)doi:10.3390/biomedicines11071899

69. Martín-Rivada Á, Palomino Pérez L, Ruiz-Sala P, et al. Diagnosis of inborn errors of metabolism within the expanded newborn screening in the Madrid region. *JIMD Rep*. Mar 2022;63(2):146-161. doi:10.1002/jmd2.12265

70. Li M, Yang Q, Yi S, Qin Z, Luo J, Fan X. Two novel mutations in DNAJC12 identified by whole-exome sequencing in a patient with mild hyperphenylalaninemia. *Mol Genet Genomic Med*. Aug 2020;8(8):e1303. doi:10.1002/mgg3.1303

71. Feng Y, Liu S, Tang C, et al. Identification of an inherited pathogenic DNAJC12 variant in a patient with hyperphenylalalinemia. *Clin Chim Acta*. Mar 2019;490:172-175. doi:10.1016/j.cca.2018.09.002

72. Navarrete R, Leal F, Vega AI, et al. Value of genetic analysis for confirming inborn errors of metabolism detected through the Spanish neonatal screening program. *Eur J Hum Genet*. Apr 2019;27(4):556-562. doi:10.1038/s41431-018-0330-0

73. Blau N, Martinez A, Hoffmann GF, Thöny B. DNAJC12 deficiency: A new strategy in the diagnosis of hyperphenylalaninemias. *Mol Genet Metab*. Jan 2018;123(1):1-5. doi:10.1016/j.ymgme.2017.11.005

74. Anikster Y, Haack TB, Vilboux T, et al. Biallelic Mutations in DNAJC12 Cause Hyperphenylalaninemia, Dystonia, and Intellectual Disability. *Am J Hum Genet*. Feb 2 2017;100(2):257-266. doi:10.1016/j.ajhg.2017.01.002

75. Zarifian Yeganeh R, Akbari Kelishomi M, Ahmadpour Jenaghard A, et al. HFE and Non-HFE Hereditary Hemochromatosis Based on Screening of 854 Individuals: 12 Years of an Iranian Experience. *Genet Test Mol Biomarkers*. Jul 2024;28(7):289-296. doi:10.1089/gtmb.2023.0764

76. Wu LY, Song ZY, Li QH, et al. Iron chelators reverse organ damage in type 4B hereditary hemochromatosis: Case reports. *Medicine (Baltimore)*. Apr 2 2021;100(13):e25258. doi:10.1097/md.0000000000025258

77. Badar S, Busti F, Ferrarini A, et al. Identification of novel mutations in hemochromatosis genes by targeted next generation sequencing in Italian patients with unexplained iron overload. *American Journal of Hematology*. 2016;91(4):420-425. doi:10.1002/ajh.24304

78. Chen SR, Yang LQ, Chong YT, et al. Novel gain of function mutation in the SLC40A1 gene associated with hereditary haemochromatosis type 4. *Intern Med J*. Jun 2015;45(6):672-6. doi:10.1111/imj.12764

79. Sham RL, Phatak PD, West C, Lee P, Andrews C, Beutler E. Autosomal dominant hereditary hemochromatosis associated with a novel ferroportin mutation and unique clinical features. *Blood Cells, Molecules, and Diseases*. 2005/03/01/ 2005;34(2):157-161. doi:<https://doi.org/10.1016/j.bcmd.2004.12.002>

80. Schimanski LM, Drakesmith H, Merryweather-Clarke AT, et al. In vitro functional analysis of human ferroportin (FPN) and hemochromatosis-associated FPN mutations. *Blood*. May 15 2005;105(10):4096-102. doi:10.1182/blood-2004-11-4502

81. Hattori A, Miyajima H, Tomosugi N, Tatsumi Y, Hayashi H, Wakusawa S. Clinicopathological study of Japanese patients with genetic iron overload syndromes. *Pathol Int*. Sep 2012;62(9):612-8. doi:10.1111/j.1440-1827.2012.02848.x

82. Kaneko Y, Miyajima H, Piperno A, et al. Measurement of serum hepcidin-25 levels as a potential test for diagnosing hemochromatosis and related disorders. *Journal of Gastroenterology*. 2010;45(11):1163-1171. doi:10.1007/s00535-010-0259-8

83. Ohba C, Osaka H, Iai M, et al. Diagnostic utility of whole exome sequencing in patients showing cerebellar and/or vermis atrophy in childhood. *Neurogenetics*. Nov 2013;14(3-4):225-32. doi:10.1007/s10048-013-0375-8

84. Frye R, Rossignol D. Metabolic disorders and abnormalities associated with autism spectrum disorder. *Journal of Pediatric Biochemistry*. 01/01 2012;2:181-191. doi:10.3233/JPB-120060

85. Dreha-Kulaczewski S, Sahoo P, Preusse M, et al. Folate receptor α deficiency - Myelin-sensitive MRI as a reliable biomarker to monitor the efficacy and long-term outcome of a new therapeutic approach. *J Inherit Metab Dis*. Mar 2024;47(2):387-403. doi:10.1002/jimd.12713

86. Wilcken B. Leukoencephalopathies associated with disorders of cobalamin and folate metabolism. *Semin Neurol*. Feb 2012;32(1):68-74. doi:10.1055/s-0032-1306389

87. Ni Q, Tang M, Chen X, et al. Fructose-1,6-bisphosphatase deficiency: estimation of prevalence in the Chinese population and analysis of genotype-phenotype association. *Frontiers in Genetics*. 2024;15doi:10.3389/fgene.2024.1296797

88. Mayatepek E, Hoffmann B, Meissner T. Inborn errors of carbohydrate metabolism. *Best Pract Res Clin Gastroenterol*. Oct 2010;24(5):607-18. doi:10.1016/j.bpg.2010.07.012

89. Pinto A, Alfadhel M, Akroyd R, et al. International practices in the dietary management of fructose 1-6 biphosphatase deficiency. *Orphanet J Rare Dis*. Jan 25 2018;13(1):21. doi:10.1186/s13023-018-0760-3

90. Kuhara T. Diagnosis of inborn errors of metabolism using filter paper urine, urease treatment, isotope dilution and gas chromatography-mass spectrometry. *J Chromatogr B Biomed Sci Appl*. Jul 5 2001;758(1):3-25. doi:10.1016/s0378-4347(01)00138-4

91. Moon S, Kim JH, Han JH, et al. Novel compound heterozygous mutations in the fructose-1,6-bisphosphatase gene cause hypoglycemia and lactic acidosis. *Metabolism*. Jan 2011;60(1):107-13. doi:10.1016/j.metabol.2009.12.021

92. Maroulis V, Agathangelidis A, Skouma A, et al. Molecular characterization of novel and rare DNA variants in patients with galactosemia. *Frontiers in Genetics*. 2023;14doi:10.3389/fgene.2023.1266353

93. Reich S, Hennermann J, Vetter B, et al. An unexpectedly high frequency of hypergalactosemia in an immigrant Bosnian population revealed by newborn screening. *Pediatr Res*. May 2002;51(5):598-601. doi:10.1203/00006450-200205000-00009

94. Kikuchi A, Wada Y, Ohura T, Kure S. The Discovery of GALM Deficiency (Type IV Galactosemia) and Newborn Screening System for Galactosemia in Japan. *Int J Neonatal Screen*. Oct 25 2021;7(4)doi:10.3390/ijns7040068

95. Schulpis KH, Thodi G, Iakovou K, et al. Clinical evaluation and mutational analysis of GALK and GALE genes in patients with galactosemia in Greece: one novel mutation and two rare cases. *J Pediatr Endocrinol Metab*. Jul 26 2017;30(7):775-779. doi:10.1515/jpem-2017-0065

96. Stroek K, Bouva MJ, Schielen P, et al. Recommendations for newborn screening for galactokinase deficiency: A systematic review and evaluation of Dutch newborn screening data. *Mol Genet Metab*. May 2018;124(1):50-56. doi:10.1016/j.ymgme.2018.03.008

97. Porta F, Pagliardini S, Pagliardini V, Ponzone A, Spada M. Newborn screening for galactosemia: a 30-year single center experience. *World J Pediatr*. May 2015;11(2):160-4. doi:10.1007/s12519-015-0017-3

98. Hennermann JB, Schadewaldt P, Vetter B, Shin YS, Mönch E, Klein J. Features and outcome of galactokinase deficiency in children diagnosed by newborn screening. *J Inherit Metab Dis*. Apr 2011;34(2):399-407. doi:10.1007/s10545-010-9270-8

99. Li Y, Ptolemy AS, Harmonay L, Kellogg M, Berry GT. Ultra fast and sensitive liquid chromatography tandem mass spectrometry based assay for galactose-1-phosphate uridylyltransferase and galactokinase deficiencies. *Mol Genet Metab*. Jan 2011;102(1):33-40. doi:10.1016/j.ymgme.2010.08.018

100. Park HD, Kim YK, Park KU, Kim JQ, Song YH, Song J. A novel c.-22T>C mutation in GALK1 promoter is associated with elevated galactokinase phenotype. *BMC Med Genet*. Mar 24 2009;10:29. doi:10.1186/1471-2350-10-29

101. Pasquali M, Yu C, Coffee B. Laboratory diagnosis of galactosemia: a technical standard and guideline of the American College of Medical Genetics and Genomics (ACMG). *Genet Med*. Jan 2018;20(1):3-11. doi:10.1038/gim.2017.172

102. Janzen N, Illsinger S, Meyer U, et al. Early cataract formation due to galactokinase deficiency: impact of newborn screening. *Arch Med Res*. Oct 2011;42(7):608-12. doi:10.1016/j.arcmed.2011.11.004

103. Kalaydjieva L, Perez-Lezaun A, Angelicheva D, et al. A Founder Mutation in the GK1 Gene Is Responsible for Galactokinase Deficiency in Roma (Gypsies). *The American Journal of Human Genetics*. 1999/11/01/ 1999;65(5):1299-1307. doi:<https://doi.org/10.1086/302611>

104. Rubio-Gozalbo ME, Derks B, Das AM, et al. Galactokinase deficiency: lessons from the GalNet registry. *Genet Med*. Jan 2021;23(1):202-210. doi:10.1038/s41436-020-00942-9

105. Wolf P, Alcalay RN, Liong C, et al. Tandem mass spectrometry assay of β-glucocerebrosidase activity in dried blood spots eliminates false positives detected in fluorescence assay. *Mol Genet Metab*. Feb 2018;123(2):135-139. doi:10.1016/j.ymgme.2017.10.011

106. Kang L, Zhan X, Gu X, Zhang H. Successful newborn screening for Gaucher disease using fluorometric assay in China. *Journal of Human Genetics*. 2017;62(8):763-768. doi:10.1038/jhg.2017.36

107. Zimran A, Revel-Vilk S, Dinur T, et al. Evaluation of Lyso-Gb1 as a biomarker for Gaucher disease treatment outcomes using data from the Gaucher Outcome Survey. *Orphanet J Rare Dis*. Jan 29 2025;20(1):43. doi:10.1186/s13023-024-03444-y

108. Vernet Machado Bressan Wilke M, Iop GD, Faqueti L, et al. A Brazilian Rare-Disease Center’s Experience with Glucosylsphingosine (lyso-Gb1) in Patients with Gaucher Disease: Exploring a Novel Correlation with IgG Levels in Plasma and a Biomarker Measurement in CSF. *International Journal of Molecular Sciences*. 2024;25(5):2870. doi:10.3390/ijms25052870

109. Dubiela P, Szymanska-Rozek P, Hasinski P, et al. Long- and Short-Term Glucosphingosine (lyso-Gb1) Dynamics in Gaucher Patients Undergoing Enzyme Replacement Therapy. *Biomolecules*. Jul 12 2024;14(7)doi:10.3390/biom14070842

110. Malinová V, Poupětová H, Řeboun M, et al. Long-Term Evaluation of Biomarkers in the Czech Cohort of Gaucher Patients. *International Journal of Molecular Sciences*. 2023;24(19):14440. doi:10.3390/ijms241914440

111. Dubiela P, Szymańska-Rożek P, Eljaszewicz A, et al. Alpha-Synuclein mRNA Level Found Dependent on L444P Variant in Carriers and Gaucher Disease Patients on Enzyme Replacement Therapy. *Biomolecules*. 2023;13(4):644. doi:10.3390/biom13040644

112. Van Baelen A, Roosens L, Devos S, Verhulst S, Eyskens F. A new multiplex analysis of glucosylsphingosine and globotriaosylsphingosine in dried blood spots by tandem mass spectrometry. *Mol Genet Metab Rep*. Dec 2023;37:100993. doi:10.1016/j.ymgmr.2023.100993

113. Dinur T, Bauer P, Beetz C, et al. Contribution of Glucosylsphingosine (Lyso-Gb1) to Treatment Decisions in Patients with Gaucher Disease. *Int J Mol Sci*. Feb 15 2023;24(4)doi:10.3390/ijms24043945

114. Dinur T, Bauer P, Beetz C, et al. Gaucher Disease Diagnosis Using Lyso-Gb1 on Dry Blood Spot Samples: Time to Change the Paradigm? *International Journal of Molecular Sciences*. 2022;23(3):1627. doi:10.3390/ijms23031627

115. Revel-Vilk S, Fuller M, Zimran A. Value of Glucosylsphingosine (Lyso-Gb1) as a Biomarker in Gaucher Disease: A Systematic Literature Review. *Int J Mol Sci*. Sep 28 2020;21(19)doi:10.3390/ijms21197159

116. Kishnani PS, Al-Hertani W, Balwani M, et al. Screening, patient identification, evaluation, and treatment in patients with Gaucher disease: Results from a Delphi consensus. *Mol Genet Metab*. Feb 2022;135(2):154-162. doi:10.1016/j.ymgme.2021.12.009

117. Tang C, Jia X, Tang F, et al. Detection of glucosylsphingosine in dried blood spots for diagnosis of Gaucher disease by LC-MS/MS. *Clin Biochem*. Jan 2021;87:79-84. doi:10.1016/j.clinbiochem.2020.10.011

118. Mak J, Cowan TM. Detecting lysosomal storage disorders by glycomic profiling using liquid chromatography mass spectrometry. *Mol Genet Metab*. Sep-Oct 2021;134(1-2):43-52. doi:10.1016/j.ymgme.2021.08.006

119. Savolainen MJ, Karlsson A, Rohkimainen S, et al. The Gaucher earlier diagnosis consensus point-scoring system (GED-C PSS): Evaluation of a prototype in Finnish Gaucher disease patients and feasibility of screening retrospective electronic health record data for the recognition of potential undiagnosed patients in Finland. *Mol Genet Metab Rep*. Jun 2021;27:100725. doi:10.1016/j.ymgmr.2021.100725

120. Cozma C, Cullufi P, Kramp G, et al. Treatment Efficiency in Gaucher Patients Can Reliably Be Monitored by Quantification of Lyso-Gb1 Concentrations in Dried Blood Spots. *Int J Mol Sci*. Jun 27 2020;21(13)doi:10.3390/ijms21134577

121. Pawliński Ł, Polus A, Tobór E, et al. MiRNA Expression in Patients with Gaucher Disease Treated with Enzyme Replacement Therapy. *Life*. 2020;11(1):2. doi:10.3390/life11010002

122. Saville JT, McDermott BK, Chin SJ, Fletcher JM, Fuller M. Expanding the clinical utility of glucosylsphingosine for Gaucher disease. *J Inherit Metab Dis*. May 2020;43(3):558-563. doi:10.1002/jimd.12192

123. Burlina AB, Polo G, Rubert L, et al. Implementation of Second-Tier Tests in Newborn Screening for Lysosomal Disorders in North Eastern Italy. *International Journal of Neonatal Screening*. 2019;5(2):24. doi:10.3390/ijns5020024

124. Hurvitz N, Dinur T, Becker-Cohen M, et al. Glucosylsphingosine (lyso-Gb1) as a Biomarker for Monitoring Treated and Untreated Children with Gaucher Disease. *International Journal of Molecular Sciences*. 2019;20(12):3033. doi:10.3390/ijms20123033

125. Polo G, Burlina AP, Ranieri E, et al. Plasma and dried blood spot lysosphingolipids for the diagnosis of different sphingolipidoses: a comparative study. *Clin Chem Lab Med*. Nov 26 2019;57(12):1863-1874. doi:10.1515/cclm-2018-1301

126. Gary SE, Ryan E, Steward AM, Sidransky E. Recent advances in the diagnosis and management of Gaucher disease. *Expert Rev Endocrinol Metab*. Mar 2018;13(2):107-118. doi:10.1080/17446651.2018.1445524

127. Spiewak J, Doykov I, Papandreou A, et al. New Perspectives in Dried Blood Spot Biomarkers for Lysosomal Storage Diseases. *Int J Mol Sci*. Jun 15 2023;24(12)doi:10.3390/ijms241210177

128. Di Rocco M, Vici CD, Burlina A, et al. Screening for lysosomal diseases in a selected pediatric population: the case of Gaucher disease and acid sphingomyelinase deficiency. *Orphanet J Rare Dis*. Jul 21 2023;18(1):197. doi:10.1186/s13023-023-02797-0

129. Ji AJ, Wang H, Ziso-Qejvanaj E, et al. A novel approach for quantitation of glucosylceramide in human dried blood spot using LC-MS/MS. *Bioanalysis*. 2015;7(12):1483-96. doi:10.4155/bio.15.77

130. Meikle PJ, Ranieri E, Simonsen H, et al. Newborn screening for lysosomal storage disorders: clinical evaluation of a two-tier strategy. *Pediatrics*. Oct 2004;114(4):909-16. doi:10.1542/peds.2004-0583

131. Gragnaniello V, Cazzorla C, Gueraldi D, et al. Light and Shadows in Newborn Screening for Lysosomal Storage Disorders: Eight Years of Experience in Northeast Italy. *International Journal of Neonatal Screening*. 2023;10(1):3. doi:10.3390/ijns10010003

132. Woo KH, Lee BH, Heo SH, et al. Allele frequency of a 24 bp duplication in exon 10 of the CHIT1 gene in the general Korean population and in Korean patients with Gaucher disease. *J Hum Genet*. May 2014;59(5):276-9. doi:10.1038/jhg.2014.16

133. Pacheco N, Uribe A. Enzymatic analysis of biomarkers for the monitoring of Gaucher patients in Colombia. *Gene*. May 25 2013;521(1):129-35. doi:10.1016/j.gene.2013.03.044

134. Giuffrida G, Markovic U, Condorelli A, et al. Glucosylsphingosine (Lyso-Gb1) as a reliable biomarker in Gaucher disease: a narrative review. *Orphanet Journal of Rare Diseases*. 2023;18(1)doi:10.1186/s13023-023-02623-7

135. Gragnaniello V, Burlina AP, Manara R, et al. Bone disease in early detected Gaucher Type I disease: A case report. *JIMD Reports*. 2022;63(5):414-419. doi:10.1002/jmd2.12314

136. Zhang W, Oehrle M, Prada CE, et al. A convenient approach to facilitate monitoring Gaucher disease progression and therapeutic response. *Analyst*. Sep 8 2017;142(18):3380-3387. doi:10.1039/c7an00938k

137. Shepherd M, Knight BA, Laskey K, McDonald TJ. Parental experiences of a diagnosis of neonatal diabetes and perceptions of newborn screening for glucose: a qualitative study. *BMJ Open*. 2020;10(11):e037312. doi:10.1136/bmjopen-2020-037312

138. McDonald TJ, Besser RE, Perry M, et al. Screening for neonatal diabetes at day 5 of life using dried blood spot glucose measurement. *Diabetologia*. Nov 2017;60(11):2168-2173. doi:10.1007/s00125-017-4383-3

139. Grünert SC, Schumann A, Baronio F, et al. Evidence for a Genotype–Phenotype Correlation in Patients with Pathogenic GLUT2 (SLC2A2) Variants. *Genes*. 2021;12(11):1785. doi:10.3390/genes12111785

140. Porta F, Pagliardini S, Pagliardini V, Ponzone A, Spada M. Newborn screening for galactosemia: a 30-year single center experience. *World Journal of Pediatrics*. 2015;11(2):160-164. doi:10.1007/s12519-015-0017-3

141. Peduto A, Spada M, Alluto A, La Dolcetta M, Ponzone A, Santer R. A novel mutation in the GLUT2 gene in a patient with Fanconi-Bickel syndrome detected by neonatal screening for galactosaemia. *J Inherit Metab Dis*. 2004;27(2):279-80. doi:10.1023/b:boli.0000028841.00833.f4

142. Yoo HW, Shin YL, Seo EJ, Kim GH. Identification of a novel mutation in the GLUT2 gene in a patient with Fanconi-Bickel syndrome presenting with neonatal diabetes mellitus and galactosaemia. *Eur J Pediatr*. Jun 2002;161(6):351-3. doi:10.1007/s00431-002-0931-y

143. Müller D, Santer R, Krawinkel M, Christiansen B, Schaub J. Fanconi-Bickel syndrome presenting in neonatal screening for galactosaemia. *J Inherit Metab Dis*. Aug 1997;20(4):607-8. doi:10.1023/a:1005375629820

144. Molares-Vila A, Corbalán-Rivas A, Carnero-Gregorio M, González-Cespón JL, Rodríguez-Cerdeira C. Biomarkers in Glycogen Storage Diseases: An Update. *Int J Mol Sci*. Apr 22 2021;22(9)doi:10.3390/ijms22094381

145. Paesold-Burda P, Baumgartner MR, Santer R, Bosshard NU, Steinmann B. Elevated serum biotinidase activity in hepatic glycogen storage disorders--a convenient biomarker. *J Inherit Metab Dis*. Nov 2007;30(6):896-902. doi:10.1007/s10545-007-0734-4

146. Overduin RJ, Gross-Valle C, Groen J, et al. Urinary tetraglucoside excretion as a biomarker in liver glycogen storage diseases. *Mol Genet Metab*. Nov 2025;146(3):109263. doi:10.1016/j.ymgme.2025.109263

147. Groen J, De Haan BM, Overduin RJ, Haijer-Schreuder AB, Derks TG, Heiner-Fokkema MR. A machine learning model accurately identifies glycogen storage disease Ia patients based on plasma acylcarnitine profiles. *Orphanet Journal of Rare Diseases*. 2025;20(1)doi:10.1186/s13023-025-03537-2

148. Bindi V, Eiroa HD, Crespo C, Martinez M, Bay L. Clinical, Biochemical and Molecular Characterization of a Cohort of Glycogen Storage Disease Type I Patients in a High Complexity Hospital in Argentina. *Journal of Inborn Errors of Metabolism and Screening*. 2021;9doi:10.1590/2326-4594-jiems-2020-0028

149. Manwaring V, Prunty H, Bainbridge K, et al. Urine analysis of glucose tetrasaccharide by HPLC; a useful marker for the investigation of patients with Pompe and other glycogen storage diseases. *J Inherit Metab Dis*. Mar 2012;35(2):311-6. doi:10.1007/s10545-011-9360-2

150. Dewulf JP, Chevalier N, Marie S, Veiga-da-Cunha M. DBS are suitable for 1,5-anhydroglucitol monitoring in GSD1b and G6PC3-deficient patients taking SGLT2 inhibitors to treat neutropenia. *Mol Genet Metab*. Nov 2023;140(3):107712. doi:10.1016/j.ymgme.2023.107712

151. Mauri A, Duse A, Palm G, et al. Molecular Genetics of GLUT1DS Italian Pediatric Cohort: 10 Novel Disease-Related Variants and Structural Analysis. *Int J Mol Sci*. Nov 4 2022;23(21)doi:10.3390/ijms232113560

152. Vulturar R, Chiș A, Pintilie S, et al. One Molecule for Mental Nourishment and More: Glucose Transporter Type 1-Biology and Deficiency Syndrome. *Biomedicines*. May 26 2022;10(6)doi:10.3390/biomedicines10061249

153. Falsaperla R, Sciuto L, La Spina L, Sciuto S, Praticò AD, Ruggieri M. Neonatal seizures as onset of Inborn Errors of Metabolism (IEMs): from diagnosis to treatment. A systematic review. *Metab Brain Dis*. Dec 2021;36(8):2195-2203. doi:10.1007/s11011-021-00798-1

154. Galosi S, Nardecchia F, Leuzzi V. Treatable Inherited Movement Disorders in Children: Spotlight on Clinical and Biochemical Features. *Mov Disord Clin Pract*. Feb 2020;7(2):154-166. doi:10.1002/mdc3.12897

155. Brockmann K. Towards a more palatable treatment for Glut1 deficiency syndrome. *Dev Med Child Neurol*. Jul 2011;53(7):580-1. doi:10.1111/j.1469-8749.2011.03946.x

156. Kwon JM. Testing for Inborn Errors of Metabolism. *Continuum (Minneap Minn)*. Feb 2018;24(1, Child Neurology):37-56. doi:10.1212/con.0000000000000563

157. Campistol J, Plecko B. Treatable newborn and infant seizures due to inborn errors of metabolism. *Epileptic Disord*. Sep 2015;17(3):229-42. doi:10.1684/epd.2015.0754

158. Zeng Q, Sang YM. Glutamate dehydrogenase hyperinsulinism: mechanisms, diagnosis, and treatment. *Orphanet J Rare Dis*. Jan 31 2023;18(1):21. doi:10.1186/s13023-023-02624-6

159. Xu A, Cheng J, Sheng H, et al. Clinical Management and Gene Mutation Analysis of Children with Congenital Hyperinsulinism in South China. *J Clin Res Pediatr Endocrinol*. Nov 22 2019;11(4):400-409. doi:10.4274/jcrpe.galenos.2019.2019.0046

160. Ferrara C, Patel P, Becker S, Stanley CA, Kelly A. Biomarkers of Insulin for the Diagnosis of Hyperinsulinemic Hypoglycemia in Infants and Children. *J Pediatr*. Jan 2016;168:212-219. doi:10.1016/j.jpeds.2015.09.045

161. Urtizberea JA, Severa G, Malfatti E. Metabolic Myopathies in the Era of Next-Generation Sequencing. *Genes (Basel)*. Apr 22 2023;14(5)doi:10.3390/genes14050954

162. Santer R, Kinner M, Steuerwald U, et al. Molecular genetic basis and prevalence of glycogen storage disease type IIIA in the Faroe Islands. *European Journal of Human Genetics*. 2001;9(5):388-391. doi:10.1038/sj.ejhg.5200632

163. Miyashita K, Fukamachi I, Nagao M, et al. An enzyme-linked immunosorbent assay for measuring GPIHBP1 levels in human plasma or serum. *J Clin Lipidol*. Jan-Feb 2018;12(1):203-210.e1. doi:10.1016/j.jacl.2017.10.022

164. Hu X, Sleeman MW, Miyashita K, et al. Monoclonal antibodies that bind to the Ly6 domain of GPIHBP1 abolish the binding of LPL. *J Lipid Res*. Jan 2017;58(1):208-215. doi:10.1194/jlr.M072462

165. Kurooka N, Eguchi J, Wada J. Role of glycosylphosphatidylinositol‐anchored high‐density lipoprotein binding protein 1 in hypertriglyceridemia and diabetes. *Journal of Diabetes Investigation*. 2023;14(10):1148-1156. doi:10.1111/jdi.14056

166. Iacocca MA, Dron JS, Hegele RA. Progress in finding pathogenic DNA copy number variations in dyslipidemia. *Curr Opin Lipidol*. Apr 2019;30(2):63-70. doi:10.1097/mol.0000000000000581

167. Schaefer EJ, Geller AS, Endress G. The biochemical and genetic diagnosis of lipid disorders. *Curr Opin Lipidol*. Apr 2019;30(2):56-62. doi:10.1097/mol.0000000000000590

168. Guay SP, Gaudet D, Brisson D. The g.-469G>A polymorphism in the GPIHBP1 gene promoter is associated with hypertriglyceridemia and has an additive effect on the risk conferred by LPL defective alleles. *Nutr Metab Cardiovasc Dis*. Apr 2013;23(4):358-65. doi:10.1016/j.numecd.2011.08.005

169. Al-Rawaf HA, Gabr SA, Iqbal A, Alghadir AH. Circulating microRNAs and hepcidin as predictors of iron homeostasis and anemia among school children: a biochemical and cross-sectional survey analysis. *Eur J Med Res*. Dec 15 2023;28(1):595. doi:10.1186/s40001-023-01579-5

170. Roetto A, Alberti F, Daraio F, et al. Exclusion of ZIRTL as candidate gene of juvenile hemochromatosis and refinement of the critical interval on 1q21. *Blood Cells Mol Dis*. Jun 2000;26(3):205-10. doi:10.1006/bcmd.2000.0297

171. Tagliaferri F, Massese M, Russo L, et al. Hepatic glycogen storage diseases type 0, VI and IX: description of an italian cohort. *Orphanet Journal of Rare Diseases*. 2022;17(1)doi:10.1186/s13023-022-02431-5

172. Arko JJ, Debeljak M, Tansek MZ, Battelino T, Groselj U. A patient with glycogen storage disease type 0 and a novel sequence variant in GYS2: a case report and literature review. *J Int Med Res*. Aug 2020;48(8):300060520936857. doi:10.1177/0300060520936857

173. Matei L, Teodorescu MI, Kozma A, Iordan Dumitru AD, Stoicescu SM, Carniciu S. PERSISTENT ASYMPTOMATIC SEVERE HYPOGLYCAEMIA DUE TO TYPE 0A GLYCOGENOSIS - GENERAL AND ORO-DENTAL ASPECTS. *Acta Endocrinol (Buchar)*. Oct-Dec 2019;15(4):526-530. doi:10.4183/aeb.2019.526

174. Kasapkara Ç S, Aycan Z, Açoğlu E, Senel S, Oguz MM, Ceylaner S. The variable clinical phenotype of three patients with hepatic glycogen synthase deficiency. *J Pediatr Endocrinol Metab*. Apr 1 2017;30(4):459-462. doi:10.1515/jpem-2016-0317

175. Nessa A, Kumaran A, Kirk R, Dalton A, Ismail D, Hussain K. Mutational analysis of the GYS2 gene in patients diagnosed with ketotic hypoglycaemia. *J Pediatr Endocrinol Metab*. 2012;25(9-10):963-7. doi:10.1515/jpem-2012-0165

176. Miwa I, Taguchi T, Asano H, et al. Low level of fasting plasma mannose in a child with glycogen storage disease type 0 (liver glycogen synthase deficiency). *Clin Chim Acta*. Jul 4 2010;411(13-14):998-9. doi:10.1016/j.cca.2010.03.024

177. Soggia AP, Correa-Giannella ML, Fortes MA, Luna AM, Pereira MA. A novel mutation in the glycogen synthase 2 gene in a child with glycogen storage disease type 0. *BMC Med Genet*. Jan 5 2010;11:3. doi:10.1186/1471-2350-11-3

178. Weinstein DA, Correia CE, Saunders AC, Wolfsdorf JI. Hepatic glycogen synthase deficiency: an infrequently recognized cause of ketotic hypoglycemia. *Mol Genet Metab*. Apr 2006;87(4):284-8. doi:10.1016/j.ymgme.2005.10.006

179. Spiegel R, Mahamid J, Orho-Melander M, Miron D, Horovitz Y. The variable clinical phenotype of liver glycogen synthase deficiency. *J Pediatr Endocrinol Metab*. Dec 2007;20(12):1339-42. doi:10.1515/jpem.2007.20.12.1339

180. Bachrach BE, Weinstein DA, Orho-Melander M, Burgess A, Wolfsdorf JI. Glycogen synthase deficiency (glycogen storage disease type 0) presenting with hyperglycemia and glucosuria: report of three new mutations. *J Pediatr*. Jun 2002;140(6):781-3. doi:10.1067/mpd.2002.124317

181. Orho M, Bosshard NU, Buist NR, et al. Mutations in the liver glycogen synthase gene in children with hypoglycemia due to glycogen storage disease type 0. *J Clin Invest*. Aug 1 1998;102(3):507-15. doi:10.1172/jci2890

182. Atay FY, Derme T, Uras N, et al. Congenital Glucose-Galactose Malabsorption in a Turkish Newborn: A Novel Mutation of Na+/Glucose Cotransporter Gene. *Dig Dis Sci*. Jan 2017;62(1):280-281. doi:10.1007/s10620-016-4348-2

183. Katz DT, Curia S, Fifi AC, Febo-Rodriguez L, Llanos-Chea A. Novel Mutation in the SLC5A1 Gene Causing Glucose-Galactose Malabsorption: First Confirmed Case From Central America. *JPGN Rep*. Nov 2023;4(4):e390. doi:10.1097/pg9.0000000000000390

184. Alamoudi LO, Alfaraidi AT, Althagafi SS, Al-Thaqafy MS, Hasosah M. Congenital Glucose-Galactose Malabsorption: A Case With a Novel SLC5A1 Mutation in a Saudi Infant. *Cureus*. Oct 2021;13(10):e18440. doi:10.7759/cureus.18440

185. Kasahara M, Maeda M, Hayashi S, Mori Y, Abe T. A missense mutation in the Na(+)/glucose cotransporter gene SGLT1 in a patient with congenital glucose-galactose malabsorption: normal trafficking but inactivation of the mutant protein. *Biochim Biophys Acta*. May 31 2001;1536(2-3):141-7. doi:10.1016/s0925-4439(01)00043-6

186. Alruwaili NW, Alshdayed F. Fructose Metabolism and Its Effect on Glucose-Galactose Malabsorption Patients: A Literature Review. *Diagnostics (Basel)*. Jan 12 2023;13(2)doi:10.3390/diagnostics13020294

187. Wang W, Wang L, Ma M. Literature review on congenital glucose-galactose malabsorption from 2001 to 2019. *J Paediatr Child Health*. Nov 2020;56(11):1779-1784. doi:10.1111/jpc.14702

188. Vallaeys L, Van Biervliet S, De Bruyn G, et al. Congenital glucose-galactose malabsorption: a novel deletion within the SLC5A1 gene. *Eur J Pediatr*. Mar 2013;172(3):409-11. doi:10.1007/s00431-012-1802-9

189. Ashraf AP, Hurst ACE, Garg A. Extreme hypertriglyceridemia, pseudohyponatremia, and pseudoacidosis in a neonate with lipoprotein lipase deficiency due to segmental uniparental disomy. *J Clin Lipidol*. May-Jun 2017;11(3):757-762. doi:10.1016/j.jacl.2017.03.015

190. Santer R, Gokçay G, Demirkol M, Gal A, Lukacs Z. Hyperchylomicronaemia due to lipoprotein lipase deficiency as a cause of false-positive newborn screening for biotinidase deficiency. *J Inherit Metab Dis*. 2005;28(2):137-40. doi:10.1007/s10545-005-7060-5

191. Maines E, Franceschi R, Rivieri F, et al. Biochemical Pattern of Methylmalonyl-CoA Epimerase Deficiency Identified in Newborn Screening: A Case Report. *Int J Neonatal Screen*. Jul 18 2024;10(3)doi:10.3390/ijns10030053

192. Manoli I, Sloan JL, Venditti CP. Isolated Methylmalonic Acidemia. In: Adam MP, Feldman J, Mirzaa GM, Pagon RA, Wallace SE, Amemiya A, eds. *GeneReviews(®)*. University of Washington, Seattle

Copyright © 1993-2025, University of Washington, Seattle. GeneReviews is a registered trademark of the University of Washington, Seattle. All rights reserved.; 1993.

193. Fernández-Lainez C, Vela-Amieva M, Reyna-Fabián M, et al. Isolated methylmalonic acidemia in Mexico: Genotypic spectrum, report of two novel MMUT variants and a possible synergistic heterozygosity effect. *Mol Genet Metab Rep*. Dec 2024;41:101155. doi:10.1016/j.ymgmr.2024.101155

194. Decru B, Lys M, Truijens K, et al. Mitochondrial HMG-CoA synthase deficiency. *Mol Genet Metab*. Jan 2025;144(1):109007. doi:10.1016/j.ymgme.2024.109007

195. Fernando M, Vijay S, Santra S, et al. Wilson's Disease and Hyperornithinemia-hyperammonemia-homocitrullinuria Syndrome in a Child: A Case Report with Lessons Learned! *Euroasian J Hepatogastroenterol*. Jul-Dec 2021;11(2):100-102. doi:10.5005/jp-journals-10018-1351

196. Auray-Blais C, Boutin M, Lavoie P, Maranda B. Neonatal Urine Screening Program in the Province of Quebec: Technological Upgrade from Thin Layer Chromatography to Tandem Mass Spectrometry. *Int J Neonatal Screen*. Mar 20 2021;7(1)doi:10.3390/ijns7010018

197. Sokoro AA, Lepage J, Antonishyn N, et al. Diagnosis and high incidence of hyperornithinemia-hyperammonemia-homocitrullinemia (HHH) syndrome in northern Saskatchewan. *J Inherit Metab Dis*. Dec 2010;33 Suppl 3:S275-81. doi:10.1007/s10545-010-9148-9

198. Wild KT, Ganetzky RD, Yudkoff M, Ierardi-Curto L. Hyperornithinemia, Hyperammonemia, and Homocitrullinuria Syndrome Causing Severe Neonatal Hyperammonemia. *JIMD Rep*. 2019;44:103-107. doi:10.1007/8904_2018_132

199. Lee HH, Poon KH, Lai CK, et al. Hyperornithinaemia-hyperammonaemia-homocitrullinuria syndrome: a treatable genetic liver disease warranting urgent diagnosis. *Hong Kong Med J*. Feb 2014;20(1):63-6. doi:10.12809/hkmj133826

200. D'Apolito O, Garofalo D, Paglia G, Zuppaldi A, Corso G. Orotic acid quantification in dried blood spots and biological fluids by hydrophilic interaction liquid chromatography tandem mass spectrometry. *Journal of Separation Science*. 2010;33(6-7):966-973. doi:<https://doi.org/10.1002/jssc.200900758>

201. Al-Dirbashi OY, Al-Hassnan ZN, Rashed MS. Determination of homocitrulline in urine of patients with HHH syndrome by liquid chromatography tandem mass spectrometry. *Anal Bioanal Chem*. Dec 2006;386(7-8):2013-7. doi:10.1007/s00216-006-0831-5

202. Conte F, Morava E, Bakar NA, et al. Phosphoglucomutase-1 deficiency: Early presentation, metabolic management and detection in neonatal blood spots. *Mol Genet Metab*. Sep-Oct 2020;131(1-2):135-146. doi:10.1016/j.ymgme.2020.08.003

203. Nolting K, Park JH, Tegtmeyer LC, et al. Limitations of galactose therapy in phosphoglucomutase 1 deficiency. *Mol Genet Metab Rep*. Dec 2017;13:33-40. doi:10.1016/j.ymgmr.2017.07.010

204. Gahr M, Schröter W. Red cell phosphoglucomutase (PGM)-deficiency: hereditary defect of the PGM1-locus. *Eur J Pediatr*. Mar 1981;136(1):63-5. doi:10.1007/bf00441713

205. Wada Y, Okamoto N. Electrospray Ionization Mass Spectrometry of Transferrin: Use of Quadrupole Mass Analyzers for Congenital Disorders of Glycosylation. *Mass Spectrom (Tokyo)*. 2022;11(1):A0103. doi:10.5702/massspectrometry.A0103

206. Gowda VK, Battina M, Shivappa SK, Benakappa N. Treatable Cause of Pancytopenia, Recurrent Infections and Refractory Epilepsy: Secondary to Hereditary Folate Malabsorption (HFM) Due to Novel Pathogenic Variant. *Indian J Pediatr*. Jun 2021;88(6):586-588. doi:10.1007/s12098-020-03548-w

207. Huddar A, Chiplunkar S, Nagappa M, et al. Child Neurology: Hereditary Folate Malabsorption. *Neurology*. Jul 6 2021;97(1):40-43. doi:10.1212/wnl.0000000000012083

208. Tan J, Li X, Guo Y, et al. Hereditary folate malabsorption with a novel mutation on SLC46A1: A case report. *Medicine (Baltimore)*. Dec 2017;96(50):e8712. doi:10.1097/md.0000000000008712

209. Torres A, Newton SA, Crompton B, et al. CSF 5-Methyltetrahydrofolate Serial Monitoring to Guide Treatment of Congenital Folate Malabsorption Due to Proton-Coupled Folate Transporter (PCFT) Deficiency. In: Zschocke J, Baumgartner M, Morava E, Patterson M, Rahman S, Peters V, eds. *JIMD Reports, Volume 24*. Springer Berlin Heidelberg; 2015:91-96.

210. Wang Q, Li X, Ding Y, Liu Y, Qin Y, Yang Y. The first Chinese case report of hereditary folate malabsorption with a novel mutation on SLC46A1. *Brain Dev*. Jan 2015;37(1):163-7. doi:10.1016/j.braindev.2014.01.010

211. Kishimoto K, Kobayashi R, Sano H, et al. Impact of folate therapy on combined immunodeficiency secondary to hereditary folate malabsorption. *Clin Immunol*. Jul 2014;153(1):17-22. doi:10.1016/j.clim.2014.03.014

212. Shin DS, Mahadeo K, Min SH, et al. Identification of novel mutations in the proton-coupled folate transporter (PCFT-SLC46A1) associated with hereditary folate malabsorption. *Mol Genet Metab*. May 2011;103(1):33-7. doi:10.1016/j.ymgme.2011.01.008

213. Borzutzky A, Crompton B, Bergmann AK, et al. Reversible severe combined immunodeficiency phenotype secondary to a mutation of the proton-coupled folate transporter. *Clin Immunol*. Dec 2009;133(3):287-94. doi:10.1016/j.clim.2009.08.006

214. Goldman ID. Hereditary Folate Malabsorption. In: Adam MP, Bick S, Mirzaa GM, Pagon RA, Wallace SE, Amemiya A, eds. *GeneReviews(®)*. University of Washington, Seattle

Copyright © 1993-2025, University of Washington, Seattle. GeneReviews is a registered trademark of the University of Washington, Seattle. All rights reserved.; 1993.

215. Zhao R, Min SH, Qiu A, et al. The spectrum of mutations in the PCFT gene, coding for an intestinal folate transporter, that are the basis for hereditary folate malabsorption. *Blood*. Aug 15 2007;110(4):1147-52. doi:10.1182/blood-2007-02-077099

216. la Marca G, Giocaliere E, Malvagia S, et al. Development and validation of a 2nd tier test for identification of purine nucleoside phosphorylase deficiency patients during expanded newborn screening by liquid chromatography-tandem mass spectrometry. *Clin Chem Lab Med*. Apr 2016;54(4):627-32. doi:10.1515/cclm-2015-0436

217. Shakerian L, Nourizadeh M, Badalzadeh M, et al. Investigating the Variation of TREC/KREC in Combined Immunodeficiencies. *Iran J Allergy Asthma Immunol*. Aug 7 2021;20(4):402-412.

218. Roifman CM, Somech R, Kavadas F, et al. Defining combined immunodeficiency. *J Allergy Clin Immunol*. Jul 2012;130(1):177-83. doi:10.1016/j.jaci.2012.04.029

219. Haijes HA, van der Ham M, Gerrits J, et al. Direct-infusion based metabolomics unveils biochemical profiles of inborn errors of metabolism in cerebrospinal fluid. *Mol Genet Metab*. May 2019;127(1):51-57. doi:10.1016/j.ymgme.2019.03.005

220. Tummolo A, Leone P, Tolomeo M, et al. Combined isobutyryl-CoA and multiple acyl-CoA dehydrogenase deficiency in a boy with altered riboflavin homeostasis. *JIMD Rep*. Jul 2022;63(4):276-291. doi:10.1002/jmd2.12292

221. Carreau C, Lenglet T, Mosnier I, et al. A juvenile ALS-like phenotype dramatically improved after high-dose riboflavin treatment. *Ann Clin Transl Neurol*. Feb 2020;7(2):250-253. doi:10.1002/acn3.50977

222. Shao X, Steiner R, Peterson AL. Newborn screening for lipid disorders. *Curr Opin Lipidol*. Jun 1 2024;35(3):149-156. doi:10.1097/mol.0000000000000928

223. Vaz FM, Jamal Y, Barto R, et al. Newborn screening for Cerebrotendinous Xanthomatosis: A retrospective biomarker study using both flow-injection and UPLC-MS/MS analysis in 20,000 newborns. *Clin Chim Acta*. Jan 15 2023;539:170-174. doi:10.1016/j.cca.2022.12.011

224. Hong X, Daiker J, Sadilek M, et al. Toward newborn screening of cerebrotendinous xanthomatosis: results of a biomarker research study using 32,000 newborn dried blood spots. *Genet Med*. Oct 2020;22(10):1606-1612. doi:10.1038/s41436-020-0846-x

225. Vaz FM, Bootsma AH, Kulik W, et al. A newborn screening method for cerebrotendinous xanthomatosis using bile alcohol glucuronides and metabolite ratios. *J Lipid Res*. May 2017;58(5):1002-1007. doi:10.1194/jlr.P075051

226. Fukao T, Mitchell G, Sass JO, Hori T, Orii K, Aoyama Y. Ketone body metabolism and its defects. *J Inherit Metab Dis*. Jul 2014;37(4):541-51. doi:10.1007/s10545-014-9704-9

227. Hori T, Yamaguchi S, Shinkaku H, et al. Inborn errors of ketone body utilization. *Pediatr Int*. 2015;57(1):41-8. doi:10.1111/ped.12585

228. Schwade JN, Endmann M, Hofmann T, Rust S, Sass JO, Rutsch F. When one disease is not enough: succinyl-CoA: 3-oxoacid coenzyme A transferase (SCOT) deficiency due to a novel mutation in OXCT1 in an infant with known phenylketonuria. *J Pediatr Endocrinol Metab*. Oct 26 2017;30(10):1121-1124. doi:10.1515/jpem-2017-0177

229. Mascarenhas S, Yeole M, Rao LP, et al. Report of a novel recurrent homozygous variant c.620A>T in three unrelated families with thiamine metabolism dysfunction syndrome 5 and review of literature. *Clin Dysmorphol*. Oct 1 2024;33(4):160-166. doi:10.1097/mcd.0000000000000490

230. Zhao D, Liu M, Jiang H, et al. Thiamine pyrophosphokinase deficiency: report of two Chinese cases and a literature review. *Front Pediatr*. 2023;11:1173787. doi:10.3389/fped.2023.1173787

231. Zhu B, Wu J, Chen G, Chen L, Yao Y. Whole Exome Sequencing Identifies a Novel Mutation of TPK1 in a Chinese Family with Recurrent Ataxia. *J Mol Neurosci*. Aug 2020;70(8):1237-1243. doi:10.1007/s12031-020-01568-x

232. Mayr JA, Freisinger P, Schlachter K, et al. Thiamine pyrophosphokinase deficiency in encephalopathic children with defects in the pyruvate oxidation pathway. *Am J Hum Genet*. Dec 9 2011;89(6):806-12. doi:10.1016/j.ajhg.2011.11.007

233. Banka S, de Goede C, Yue WW, et al. Expanding the clinical and molecular spectrum of thiamine pyrophosphokinase deficiency: a treatable neurological disorder caused by TPK1 mutations. *Mol Genet Metab*. Dec 2014;113(4):301-6. doi:10.1016/j.ymgme.2014.09.010

234. Li D, Song J, Li X, et al. Eleven novel mutations and clinical characteristics in seven Chinese patients with thiamine metabolism dysfunction syndrome. *Eur J Med Genet*. Oct 2020;63(10):104003. doi:10.1016/j.ejmg.2020.104003

235. Schubert Baldo M, Vilarinho L. Molecular basis of Leigh syndrome: a current look. *Orphanet J Rare Dis*. Jan 29 2020;15(1):31. doi:10.1186/s13023-020-1297-9

236. Li X, Huang Z, Chen Y, et al. Case report of two affected siblings in a family with thiamine metabolism dysfunction syndrome 5: a rare, but treatable neurodegenerative disease. *BMC Neurol*. Sep 29 2022;22(1):373. doi:10.1186/s12883-022-02887-9

237. Alfadhel M, Umair M, Almuzzaini B, et al. Targeted SLC19A3 gene sequencing of 3000 Saudi newborn: a pilot study toward newborn screening. *Ann Clin Transl Neurol*. Oct 2019;6(10):2097-2103. doi:10.1002/acn3.50898

238. Ygberg S, Naess K, Eriksson M, et al. Biotin and Thiamine Responsive Basal Ganglia Disease--A vital differential diagnosis in infants with severe encephalopathy. *Eur J Paediatr Neurol*. May 2016;20(3):457-61. doi:10.1016/j.ejpn.2016.01.009

239. Wassenberg T, Geurtz BPH, Monnens L, Wevers RA, Willemsen MA, Verbeek MM. Blood, urine and cerebrospinal fluid analysis in TH and AADC deficiency and the effect of treatment. *Mol Genet Metab Rep*. Jun 2021;27:100762. doi:10.1016/j.ymgmr.2021.100762

240. Bijarnia-Mahay S, Jain V, Thöny B. Tyrosine hydroxylase deficiency-Clinical insights and a novel deletion in TH gene in an Indian patient. *JIMD Rep*. May 2020;53(1):12-15. doi:10.1002/jmd2.12111

241. Billington CJ, Jr., Rayannavar A, Tryon R, et al. Prognostication and Biomarker Potential of C26:0 Lysophosphatidylcholine in Adrenoleukodystrophy. *JAMA Pediatr*. Apr 1 2025;179(4):465-467. doi:10.1001/jamapediatrics.2024.6774

242. Tang C, Tang F, Cai Y, et al. A pilot study of newborn screening for X-linked adrenoleukodystrophy based on liquid chromatography-tandem mass spectrometry method for detection of C26:0-lysophosphatidylcholine in dried blood spots: Results from 43,653 newborns in a southern Chinese population. *Clin Chim Acta*. Jan 1 2024;552:117653. doi:10.1016/j.cca.2023.117653

243. Morales-Romero B, González de Aledo-Castillo JM, Fernández Sierra C, et al. Plasma C24:0- and C26:0-lysophosphatidylcholines are reliable biomarkers for the diagnosis of peroxisomal β-oxidation disorders. *J Lipid Res*. Mar 2024;65(3):100516. doi:10.1016/j.jlr.2024.100516

244. Videbæk C, Melgaard L, Lund AM, Grønborg SW. Newborn screening for adrenoleukodystrophy: International experiences and challenges. *Mol Genet Metab*. Dec 2023;140(4):107734. doi:10.1016/j.ymgme.2023.107734

245. Kilgore MB, Platis D, Lim T, et al. Development of a Universal Second-Tier Newborn Screening LC-MS/MS Method for Amino Acids, Lysophosphatidylcholines, and Organic Acids. *Anal Chem*. Feb 14 2023;95(6):3187-3194. doi:10.1021/acs.analchem.2c03098

246. Baker CV, Cady Keller A, Lutz R, et al. Newborn Screening for X-Linked Adrenoleukodystrophy in Nebraska: Initial Experiences and Challenges. *Int J Neonatal Screen*. Apr 26 2022;8(2)doi:10.3390/ijns8020029

247. Teber TA, Conti BJ, Haynes CA, Hietala A, Baker MW. Newborn Screen for X-Linked Adrenoleukodystrophy Using Flow Injection Tandem Mass Spectrometry in Negative Ion Mode. *Int J Neonatal Screen*. Apr 14 2022;8(2)doi:10.3390/ijns8020027

248. Kettwig M, Klemp H, Nessler S, et al. Targeted metabolomics revealed changes in phospholipids during the development of neuroinflammation in Abcd1(tm1Kds) mice and X-linked adrenoleukodystrophy patients. *J Inherit Metab Dis*. Sep 2021;44(5):1174-1185. doi:10.1002/jimd.12389

249. Natarajan A, Christopher R, Netravathi M, Bhat M, Chandra SR. Liquid chromatography-tandem mass spectrometry method for estimation of a panel of lysophosphatidylcholines in dried blood spots for screening of X-linked adrenoleukodystrophy. *Clin Chim Acta*. Oct 2018;485:305-310. doi:10.1016/j.cca.2018.07.007

250. Hong X, Kumar AB, Ronald Scott C, Gelb MH. Multiplex tandem mass spectrometry assay for newborn screening of X-linked adrenoleukodystrophy, biotinidase deficiency, and galactosemia with flexibility to assay other enzyme assays and biomarkers. *Mol Genet Metab*. Jun 2018;124(2):101-108. doi:10.1016/j.ymgme.2018.03.012

251. Mashima R, Tanaka M, Sakai E, et al. A selective detection of lysophosphatidylcholine in dried blood spots for diagnosis of adrenoleukodystrophy by LC-MS/MS. *Mol Genet Metab Rep*. Jun 2016;7:16-9. doi:10.1016/j.ymgmr.2016.02.007

252. Haynes CA, De Jesús VR. Simultaneous quantitation of hexacosanoyl lysophosphatidylcholine, amino acids, acylcarnitines, and succinylacetone during FIA-ESI-MS/MS analysis of dried blood spot extracts for newborn screening. *Clin Biochem*. Jan 2016;49(1-2):161-5. doi:10.1016/j.clinbiochem.2015.09.011

253. Haynes CA, De Jesús VR. The stability of hexacosanoyl lysophosphatidylcholine in dried-blood spot quality control materials for X-linked adrenoleukodystrophy newborn screening. *Clin Biochem*. Jan 2015;48(1-2):8-10. doi:10.1016/j.clinbiochem.2014.10.001

254. Van De Beek M-C, Dijkstra IME, Van Lenthe H, et al. C26:0-Carnitine Is a New Biomarker for X-Linked Adrenoleukodystrophy in Mice and Man. *PLOS ONE*. 2016;11(4):e0154597. doi:10.1371/journal.pone.0154597

255. Huffnagel IC, van de Beek MC, Showers AL, et al. Comparison of C26:0-carnitine and C26:0-lysophosphatidylcholine as diagnostic markers in dried blood spots from newborns and patients with adrenoleukodystrophy. *Mol Genet Metab*. Dec 2017;122(4):209-215. doi:10.1016/j.ymgme.2017.10.012

256. Wu C, Iwamoto T, Igarashi J, et al. Application of a diagnostic methodology by quantification of 26:0 lysophosphatidylcholine in dried blood spots for Japanese newborn screening of X-linked adrenoleukodystrophy. *Mol Genet Metab Rep*. Sep 2017;12:115-118. doi:10.1016/j.ymgmr.2017.06.004

257. Tian GL, Xu F, Jiang K, Wang YM, Ji W, Zhuang YP. Evaluation of a panel of very long-chain lysophosphatidylcholines and acylcarnitines for screening of X-linked adrenoleukodystrophy in China. *Clin Chim Acta*. Apr 2020;503:157-162. doi:10.1016/j.cca.2020.01.016

258. Kemp S, Huffnagel IC, Linthorst GE, Wanders RJ, Engelen M. Adrenoleukodystrophy - neuroendocrine pathogenesis and redefinition of natural history. *Nat Rev Endocrinol*. Oct 2016;12(10):606-15. doi:10.1038/nrendo.2016.90

259. Bonilla Guerrero R, Wolfe LA, Payne N, et al. Essential fatty acid profiling for routine nutritional assessment unmasks adrenoleukodystrophy in an infant with isovaleric acidaemia. *J Inherit Metab Dis*. Dec 2008;31 Suppl 2:S453-6. doi:10.1007/s10545-008-1039-y

260. Huffnagel IC, Laheji FK, Aziz-Bose R, et al. The Natural History of Adrenal Insufficiency in X-Linked Adrenoleukodystrophy: An International Collaboration. *J Clin Endocrinol Metab*. Jan 1 2019;104(1):118-126. doi:10.1210/jc.2018-01307

261. Damiano R, Della Bona M, Procopio E, Guerrini R, Bettiol A, la Marca G. Inclusion of pyridoxine dependent epilepsy in expanded newborn screening programs by tandem mass spectrometry: set up of first and second tier tests. *Clin Chem Lab Med*. Jun 26 2025;63(7):1344-1353. doi:10.1515/cclm-2024-1230

262. Pauly K, Woontner M, Abdenur JE, et al. Feasibility of newborn screening for pyridoxine-dependent epilepsy. *Mol Genet Metab*. Jan 2025;144(1):109002. doi:10.1016/j.ymgme.2024.109002

263. Engelke UF, van Outersterp RE, Merx J, et al. Untargeted metabolomics and infrared ion spectroscopy identify biomarkers for pyridoxine-dependent epilepsy. *J Clin Invest*. Aug 2 2021;131(15)doi:10.1172/jci148272

264. Coughlin CR, 2nd, Tseng LA, van Karnebeek CDM. A case for newborn screening for pyridoxine-dependent epilepsy. *Cold Spring Harb Mol Case Stud*. Feb 2022;8(2)doi:10.1101/mcs.a006197

265. Wempe MF, Kumar A, Kumar V, et al. Identification of a novel biomarker for pyridoxine-dependent epilepsy: Implications for newborn screening. *J Inherit Metab Dis*. May 2019;42(3):565-574. doi:10.1002/jimd.12059

266. Jung S, Tran NT, Gospe SM, Jr., Hahn SH. Preliminary investigation of the use of newborn dried blood spots for screening pyridoxine-dependent epilepsy by LC-MS/MS. *Mol Genet Metab*. Nov 2013;110(3):237-40. doi:10.1016/j.ymgme.2013.07.017

267. Mathew EM, Moorkoth S, Lewis L, Rao P. Biomarker Profiling for Pyridoxine Dependent Epilepsy in Dried Blood Spots by HILIC-ESI-MS. *Int J Anal Chem*. 2018;2018:2583215. doi:10.1155/2018/2583215

268. Xue J, Wang J, Gong P, et al. Simultaneous quantification of alpha-aminoadipic semialdehyde, piperideine-6-carboxylate, pipecolic acid and alpha-aminoadipic acid in pyridoxine-dependent epilepsy. *Sci Rep*. Aug 6 2019;9(1):11371. doi:10.1038/s41598-019-47882-2

269. Judy RL, Reynolds JL, Jnah AJ. Identifying Metabolic Diseases That Precipitate Neonatal Seizures. *Neonatal Netw*. May 1 2024;43(3):139-147. doi:10.1891/nn-2023-0048

270. Damiano R, Della Bona M, Procopio E, et al. Determination of new biomarkers for diagnosis of pyridoxine dependent epilepsy in human plasma and urine by liquid chromatography-mass spectrometry. *Clin Chim Acta*. Feb 1 2025;567:120111. doi:10.1016/j.cca.2024.120111

271. van Outersterp RE, Engelke UFH, Merx J, et al. Metabolite Identification Using Infrared Ion Spectroscopy─Novel Biomarkers for Pyridoxine-Dependent Epilepsy. *Anal Chem*. Nov 23 2021;93(46):15340-15348. doi:10.1021/acs.analchem.1c02896

272. Jeridi C, Rachdi A, Nabli F, et al. Genetic heterogeneity within a consanguineous family involving TTPA and SETX genes. *J Neurogenet*. Sep-Dec 2023;37(4):124-130. doi:10.1080/01677063.2023.2281916

273. Elkamil A, Johansen KK, Aasly J. Ataxia with vitamin e deficiency in norway. *J Mov Disord*. Jan 2015;8(1):33-6. doi:10.14802/jmd.14030

274. Manor D, Morley S. The alpha-tocopherol transfer protein. *Vitam Horm*. 2007;76:45-65. doi:10.1016/s0083-6729(07)76003-x

275. Zhang LW, Liu B, Peng DT. Clinical and genetic study of ataxia with vitamin E deficiency: A case report. *World J Clin Cases*. Aug 16 2022;10(23):8271-8276. doi:10.12998/wjcc.v10.i23.8271

276. Gonzales E, Cresteil D, Baussan C, Dabadie A, Gerhardt MF, Jacquemin E. SRD5B1 (AKR1D1) gene analysis in delta(4)-3-oxosteroid 5beta-reductase deficiency: evidence for primary genetic defect. *J Hepatol*. Apr 2004;40(4):716-8. doi:10.1016/j.jhep.2003.12.024

277. Gardin A, Ruiz M, Beime J, et al. ∆(4)-3-oxo-5β-reductase deficiency: favorable outcome in 16 patients treated with cholic acid. *Orphanet J Rare Dis*. Dec 7 2023;18(1):383. doi:10.1186/s13023-023-02984-z

278. Zhao J, Qiu YL, Wang L, et al. Recurrent AKR1D1 c.580-13T>A Variant: A Cause of Δ(4)-3-Oxosteroid-5β-Reductase Deficiency. *J Mol Diagn*. Apr 2023;25(4):227-233. doi:10.1016/j.jmoldx.2023.01.004

279. Zhang MH, Setchell KD, Zhao J, Gong JY, Lu Y, Wang JS. Δ4-3-oxosteroid-5β-reductase deficiency: Responses to oral bile acid therapy and long-term outcomes. *World J Gastroenterol*. Feb 21 2019;25(7):859-869. doi:10.3748/wjg.v25.i7.859

280. Yanagi T, Mizuochi T, Homma K, et al. Distinguishing primary from secondary Δ(4) -3-oxosteroid 5β-reductase (SRD5B1, AKR1D1) deficiency by urinary steroid analysis. *Clin Endocrinol (Oxf)*. Mar 2015;82(3):346-51. doi:10.1111/cen.12596

281. Kimura A, Mori J, Pham AN, et al. Healthy Patients With AKR1D1 Mutation Not Requiring Primary Bile Acid Therapy: A Case Series. *JPGN Rep*. Nov 2023;4(4):e372. doi:10.1097/pg9.0000000000000372

**Appendix B**

*1. CLIR database search strategy*

To identify biomarkers for IMD in the CLIR database (Biochemical Genetics Laboratory et al.), the ‘*Productivity Tools’* tab was used, followed by the ‘*Plot by Condition’* tab. Each of the IMD corresponding to the 95 treatable IMD (100 genes) from the NGSf4NBS list (Veldman et al., 2024) was selected via the ‘*Conditions*’ index. Under the tab ‘*Select Marker(s)’*, all boxes with biomarkers (including ratios and others) were checked, and under the tab ‘*Additional Options’,* the box with ‘*Show Informative Markers Only*?’ was checked. The settings *‘View All Markers’*, *‘Unadjusted Values’*, and *‘Multiple of Median (positive values only)’* were left unadjusted. By clicking on *‘Show Chart’,* a chart with box plots for all biomarkers within the database was created. To select only those biomarkers that are 100% sensitive (not overlapping with the reference population), the ‘*Maximum %ile Overlap*’ slider bar on the right side of the chart was adjusted to 0.0%. The chart with box plots of each IMD was exported into a PDF file and saved in a shared database.

Data on the number of cases included in the database was obtained by referring to the *‘Productivity Tools’* tab, followed by *‘Case Count per Condition’*, and then per IMD by clicking on *‘Show Chart’*.

*2. Search strategies for DBS biomarkers in PubMed/Medline and Embase*

*("Dried Blood Spot Testing" OR "Dried Blood Spot*"[All fields] OR "DBS"[All fields] OR "Dried Blood Spot Testing"[All fields] OR "Neonatal Screening"[Mesh] OR "Neonatal Screening"[All fields] OR "Newborn screening"[All fields] OR "newborn infant screen*"[All fields] OR "heel prick*"[All fields] OR "heel stick"[All fields] OR "Guthrie's test"[All fields])*

AND

*(“IMD name (if available MeSH terms), synonyms/alternative IMD names, IMD abbreviations, the name of the gene(s), Online Mendelian Inheritance in Man (OMIM) numbers locus + phenotype (found on IEMbase, OMIM and PubMed MeSH entry terms”)*

If the list of retrieved publications was too large (>75 reports), a third more-specific query was added: *("Biomarkers"[Mesh] OR "biomarker*"[All fields] OR "Biomarkers/blood"[Mesh] OR "Biomarkers/chemistry"[Mesh] OR "Blood biomarker"[All fields])*

Using the query translator tool in Embase, the PubMed query was translated into a search strategy that can be used for Embase.

3. Extended search for biomarkers in other biological material (non-DBS)

The search terms used were a combination of: *“IMD name (if available MeSH terms)”,* *“synonyms/alternative IMD names”*, “*IMD abbreviations”,* and “*gene name”.*

In too many results were displayed, the following search terms and Mesh terms were added: *“Urine”, “Urinalysis”, “Serum”, “Plasma”, “Blood”, “Erythrocytes”, “red blood cells”,* “*Lymphocytes”, “Leukocytes”, “Stool”, “Feces”, “Cerebrospinal Fluid”, “Saliva”, “Biopsy”,* “*Connective Tissue Cells", "Fibroblasts”, “Bone Marrow Cells”, “Myeloid Cells”, “Muscle Cells”, "Epithelial Cells”, “Epidermal Cells”, “Endocrine Cells”.*

Specific search strings for each IMD and retrieved PubMed hits can be requested from the authors.

**Appendix C**

| **Section and Topic** | **Item #** | **Checklist item** | **Location where item is reported** |
| --- | --- | --- | --- |
| **TITLE** | | |  |
| Title | 1 | Identify the report as a systematic review. | N.A. |
| **ABSTRACT** | | |  |
| Abstract | 2 | See the PRISMA 2020 for Abstracts checklist. | P2 |
| **INTRODUCTION** | | |  |
| Rationale | 3 | Describe the rationale for the review in the context of existing knowledge. | P4−7 Introduction |
| Objectives | 4 | Provide an explicit statement of the objective(s) or question(s) the review addresses. | P6 last paragraph – P7 first paragraph  *At present (…) identified previously* |
| **METHODS** | | |  |
| Eligibility criteria | 5 | Specify the inclusion and exclusion criteria for the review and how studies were grouped for the syntheses. | P11 *Here, we (..) using DBS.*  And,  P11 2^nd^ paragraph: *If for (…) Step 3 (see below)* |
| Information sources | 6 | Specify all databases, registers, websites, organisations, reference lists and other sources searched or consulted to identify studies. Specify the date when each source was last searched or consulted. | P8−12 *Methods 2.2.−2.5.,*  Figures 1 & 3 |
| Search strategy | 7 | Present the full search strategies for all databases, registers and websites, including any filters and limits used. | Appendix B |
| Selection process | 8 | Specify the methods used to decide whether a study met the inclusion criteria of the review, including how many reviewers screened each record and each report retrieved, whether they worked independently, and if applicable, details of automation tools used in the process. | P7−12 *Methods 2.1.−2.5*.,  P12 *Methods 2.6.: Two independent (…) PRISMA 2020 guidelines.* |
| Data collection process | 9 | Specify the methods used to collect data from reports, including how many reviewers collected data from each report, whether they worked independently, any processes for obtaining or confirming data from study investigators, and if applicable, details of automation tools used in the process. | P12 *Methods 2.6.: Two independent (…) PRISMA 2020 guidelines.* |
| Data items | 10a | List and define all outcomes for which data were sought. Specify whether all results that were compatible with each outcome domain in each study were sought (e.g. for all measures, time points, analyses), and if not, the methods used to decide which results to collect. | P11 Methods 2.4.  *A comprehensive PubMed (…) a classification* |
|  | 10b | List and define all other variables for which data were sought (e.g. participant and intervention characteristics, funding sources). Describe any assumptions made about any missing or unclear information. | P7−12 Methods *2.1.*−*2.5.,*  P12 *Methods 2.6: search strategies (…) citation bias.* |
| Study risk of bias assessment | 11 | Specify the methods used to assess risk of bias in the included studies, including details of the tool(s) used, how many reviewers assessed each study and whether they worked independently, and if applicable, details of automation tools used in the process. | P12 *Methods 2.6.* |
| Effect measures | 12 | Specify for each outcome the effect measure(s) (e.g. risk ratio, mean difference) used in the synthesis or presentation of results. | N.A. / Effect measures are mentioned in P7−8 *Based on (…) available (Appendix A)* |
| Synthesis methods | 13a | Describe the processes used to decide which studies were eligible for each synthesis (e.g. tabulating the study intervention characteristics and comparing against the planned groups for each synthesis (item #5)). | All identified studies and the process are described in Appendix A, Figure 1 and Figure 3 |
|  | 13b | Describe any methods required to prepare the data for presentation or synthesis, such as handling of missing summary statistics, or data conversions. | Appendix B |
|  | 13c | Describe any methods used to tabulate or visually display results of individual studies and syntheses. | N.A. |
|  | 13d | Describe any methods used to synthesize results and provide a rationale for the choice(s). If meta-analysis was performed, describe the model(s), method(s) to identify the presence and extent of statistical heterogeneity, and software package(s) used. | N.A. |
|  | 13e | Describe any methods used to explore possible causes of heterogeneity among study results (e.g. subgroup analysis, meta-regression). | N.A. |
|  | 13f | Describe any sensitivity analyses conducted to assess robustness of the synthesized results. | N.A. |
| Reporting bias assessment | 14 | Describe any methods used to assess risk of bias due to missing results in a synthesis (arising from reporting biases). | P12 *Methods 2.6.* |
| Certainty assessment | 15 | Describe any methods used to assess certainty (or confidence) in the body of evidence for an outcome. | P12 *Methods 2.6.* |
| **RESULTS** | | |  |
| Study selection | 16a | Describe the results of the search and selection process, from the number of records identified in the search to the number of studies included in the review, ideally using a flow diagram. | Figure 3 |
|  | 16b | Cite studies that might appear to meet the inclusion criteria, but which were excluded, and explain why they were excluded. | These are non-DBS biomarker data (n=100)  P14−15 Results 3.2.1.: *Of these 206 (…) documented in Step 3,*  Appendix A (in a separate non-DBS column) |
| Study characteristics | 17 | Cite each included study and present its characteristics. | Appendix A (study characteristics are not presented, due to the quantity of included studies) |
| Risk of bias in studies | 18 | Present assessments of risk of bias for each included study. | N.A. |
| Results of individual studies | 19 | For all outcomes, present, for each study: (a) summary statistics for each group (where appropriate) and (b) an effect estimate and its precision (e.g. confidence/credible interval), ideally using structured tables or plots. | N.A. |
| Results of syntheses | 20a | For each synthesis, briefly summarise the characteristics and risk of bias among contributing studies. | N.A. |
|  | 20b | Present results of all statistical syntheses conducted. If meta-analysis was done, present for each the summary estimate and its precision (e.g. confidence/credible interval) and measures of statistical heterogeneity. If comparing groups, describe the direction of the effect. | N.A. |
|  | 20c | Present results of all investigations of possible causes of heterogeneity among study results. | Appendix A |
|  | 20d | Present results of all sensitivity analyses conducted to assess the robustness of the synthesized results. | N.A. |
| Reporting biases | 21 | Present assessments of risk of bias due to missing results (arising from reporting biases) for each synthesis assessed. | Out of the scope of our study |
| Certainty of evidence | 22 | Present assessments of certainty (or confidence) in the body of evidence for each outcome assessed. | P14−15 Results 3.2.1., P17 Results 3.3.,  Figure 3 and 4. |
| **DISCUSSION** | | |  |
| Discussion | 23a | Provide a general interpretation of the results in the context of other evidence. | P18−19 *Discussion: This study (…) NBS tests,*  *P19*−*24 Discussion:*  *Overall, genetic-based (…) substantially expands.* |
|  | 23b | Discuss any limitations of the evidence included in the review. | P24−25 A limitation (…) our study |
|  | 23c | Discuss any limitations of the review processes used. | P24−25 A limitation (…) our study |
|  | 23d | Discuss implications of the results for practice, policy, and future research. | P23−24 Currently, practical (…) substantially expands.  P24 Regardless of (…) implementation studies  P25 In addition (…) actively pursued |
| **OTHER INFORMATION** | | |  |
| Registration and protocol | 24a | Provide registration information for the review, including register name and registration number, or state that the review was not registered. | The review was not registered |
|  | 24b | Indicate where the review protocol can be accessed, or state that a protocol was not prepared. | A protocol was not prepared |
|  | 24c | Describe and explain any amendments to information provided at registration or in the protocol. | N.A. |
| Support | 25 | Describe sources of financial or non-financial support for the review, and the role of the funders or sponsors in the review. | See funding statement and author contributions P26 |
| Competing interests | 26 | Declare any competing interests of review authors. | See Conflict of Interest P26 |
| Availability of data, code and other materials | 27 | Report which of the following are publicly available and where they can be found: template data collection forms; data extracted from included studies; data used for all analyses; analytic code; any other materials used in the review. | All relevant data is provided in the manuscript and appendices. Search strings can be obtained from the authors |
